# Supplementary material for: Pilot protocol for the Parent and Infant Inter(X)action Intervention (PIXI) feasibility study
Source: PLoS One. 2023 May 4;18(5):e0270169. doi: 10.1371/journal.pone.0270169 (PMC10159119; doi:10.1371/journal.pone.0270169)
Supplement: S1 File — (PDF) [file pone.0270169.s002.pdf]

THE UNIVERSITY of NORTH CAROLINA at CHAPEL HILL

OFFICE OF HUMAN RESEARCH ETHICS
720 Martin Luther King, Jr. Blvd.
Bldg. 385, 2nd Floor
CB #7097
Chapel Hill, NC 27599-7097
(919) 966-3113
Web site: ohre.unc.edu
Federalwide Assurance (FWA) #4801

To: Lauren Turner-Brown,
Psychiatry - TEACCH Division

From: Biomedical IRB

Approval Date: 11/15/2018
Expiration Date of Approval: 11/14/2019
RE: Notice of IRB Approval by Expedited Review (under 45 CFR 46.110)
Submission Type: Initial
Expedited Category: 5.Existing or non-research data,6.Voice/image research recordings,7.Surveys/interviews/focus groups
Study #: 18-2079

Study Title: Pilot Study: A Parent-Infant fragile X Intervention (PiXI)

This submission has been approved by the IRB for the period indicated. It has been determined that the risk involved in this research is no more than minimal.

Study Description:

Purpose: The objective is to develop and test, through an iterative process, an intervention to address and support the development of infants with a confirmed diagnosis of fragile X syndrome (FXS). The proposed project will capitalize and expand upon existing empirically based interventions designed to improve outcomes for infants with suspected developmental and social delays.

Participants: Participants will be newborns diagnosed with FXS identified through Early Check (IRB #18-0009), a voluntary research program in which newborns will be screened for a carefully selected panel of conditions.

Procedures (methods): The intervention, called Parent-infant fragile X Intervention (PiXI) will consist of two phases. Phase 1 will include parent education about early infant development and FXS. Phase 2 includes direct parent coaching around parent-child interaction based on an empirically based parent-mediated early intervention, the Intervention within the British Autism study of Infant Siblings- Video-feedback Intervention to promote Positive parenting (i-BASIS-VIPP), and repeated comprehensive assessments of family and child functioning.

Study Regulatory and other findings:

This research, which involves children, meets criteria at 45 CFR 46.404 and/or 21 CFR 50.51 (research involving no greater than minimal risk). The IRB has determined that the study-specific rationale provided by the investigator in application section A.2.A is sufficient to justify this finding. Permission of one parent or guardian is sufficient.

Investigator's Responsibilities:

Federal regulations require that all research be reviewed at least annually. It is the Principal Investigator's responsibility to submit for renewal and obtain approval before the expiration date. You may not continue any research activity beyond the expiration date without IRB approval. Failure to receive approval for

continuation before the expiration date will result in automatic termination of the approval for this study on the expiration date.

Your approved consent forms and other documents are available online at http://apps.research.unc.edu/irb/index.cfm?event=home.dashboard.irbStudyManagement&irb_id=18-2079.

You are required to obtain IRB approval for any changes to any aspect of this study before they can be implemented. Any unanticipated problem involving risks to subjects or others (including adverse events reportable under UNC-Chapel Hill policy) should be reported to the IRB using the web portal at http://irbis.unc.edu.

Please be aware that additional approvals may still be required from other relevant authorities or "gatekeepers" (e.g., school principals, facility directors, custodians of records).

The current data security level determination is Level II. Any changes in the data security level need to be discussed with the relevant IT official. If data security level II and III, consult with your IT official to develop a data security plan. Data security is ultimately the responsibility of the Principal Investigator.

This study was reviewed in accordance with federal regulations governing human subjects research, including those found at 45 CFR 46 (Common Rule), 45 CFR 164 (HIPAA), 21 CFR 50 & 56 (FDA), and 40 CFR 26 (EPA), where applicable.

IRB Authorization Agreements have been executed for the UNC IRB to provide IRB review and continuing oversight for human subjects research performed for this study at the organizations identified below:

- Research Triangle Institute (RTI International)

A participating organization not included on this list may not proceed with human subjects research for this study until the UNC IRB approval for the organization is granted. Please note that approval for additional participating organizations requires a modification to the study.

CC:
MaryKate Frisch, TEACCH Autism Program
Casey Okoniewski
Melissa Raspa
Anne Wheeler
