# Supplementary material for: Pilot protocol for the Parent and Infant Inter(X)action Intervention (PIXI) feasibility study
Source: PLoS One. 2023 May 4;18(5):e0270169. doi: 10.1371/journal.pone.0270169 (PMC10159119; doi:10.1371/journal.pone.0270169)
Supplement: S2 File — (PDF) [file pone.0270169.s003.pdf]

Post Approval Submissions

Modification Information

To modify an approved study, edit the individual answers that make up the application. The questions below are intended solely for the IRB to have a summary statement of your requested action. The modifications cannot be processed until the actual changes have been made throughout the application.

1. Provide a brief non-technical summary of any changes you will be making to the study (i.e., study application, project personnel, and/or study documents.) The text you enter here will be reproduced in the IRB approval document, and should contain the details that you and/or your sponsor find relevant (e.g., master protocol/amendment version number and date). Typical summaries are 50-100 words. PLEASE NOTE: THIS SECTION MAY BE EDITED BY THE IRB FOR CLARITY OR LENGTH.

   The following changes are requested in this submission:

   1. A participating family has asked that both parents be allowed to take part in the study. Allowing both parents to participate will likely improve the outcomes of the intervention, thus we would like to allow both parents to participate if both ask to participate. We will ask the 2nd parent to sign a 2nd consent form and ask them to complete the parent report surveys as well.

   2. We are adding a virtual adaptation of the DAYC-2 (protocol has been uploaded) to offer participants more flexibility during telehealth appointments. We will be conducting the assessment with participants in addition to select components of PANDABox, and will only be administered if participants are not engaging in an in-person assessment, or completing the DP-4. Incorporating this measure will not add to the current battery but complement or supplement it accordingly The addition of this measure will not alter the assessment times dscribed in the current consent form. Because the time estimation is still accurate and the individual measures are not listed in the consent form, we do not plan to re-consent paricipants currently enrolled.

2. Is this study in Data Analysis only (i.e. enrollment, intervention and follow-up are complete)?

   No

Total number of subjects enrolled to date:

30

Is this study currently open to the enrollment of new subjects?

Yes

Total number of subjects actively participating (i.e., Total number of subjects involved in the interventional part of this study. If the study is limited to data collection (e.g., surveys, questionnaires, collection of data from existing records), enter ‘0’:

6

3. Do you have plans to re-consent subjects as a result of this modification?

   No

4. Is this modification being submitted in response to Promptly Reportable Information?

   No

5. Have the risks as described in A.6., consent form, or any other study document changed?
   This may include new risks not previously listed, changes in frequency of known risks, or removal of previously listed risks.

   No

Continuing with Modifications

Click the "save and continue" button to access your existing application.
You may make any changes to the application that you are requesting at this time.

General Information

1. General Information

1. Project Title

   Pilot Study: A Parent-Infant Interaction Intervention (PIXI)

2. **Brief Summary**. Provide a **brief non-technical description** of the study, which will be used in IRB documentation as a description of the study. Typical summaries are 50-100 words. Please reply to each item below, retaining the subheading labels already in place, so that reviewers can readily identify the content. PLEASE NOTE: THIS SECTION MAY BE EDITED BY THE IRB FOR CLARITY OR LENGTH.

   Purpose: The objective is to develop and test, through an iterative process, an intervention to address and support the development of infants with a confirmed diagnosis of neurogenetic disorders that leave individuals at risk for developmental delays or intellectual and developmental disabilities (e.g., fragile X syndrome, Duchesne muscular dystrophy).. The proposed project will capitalize and expand upon existing empirically based interventions designed to improve outcomes for infants with suspected developmental and social delays.

   Participants: Participants will be infants with a confirmed diagnosis of a neurogentic disorder within the first year of life and one or both parents. The majority of participants will be identified through Early Check (IRB #18-0009), a voluntary research program in which newborns will be screened for a carefully selected panel of conditions. Additionally, families can self-refer and if they meet eligibility requirements, may enroll in the intervention.

   Procedures (methods): The intervention, called Parent-infant Interaction Intervention (PIXI) will consist of two phases. Phase 1 will include parent education about early infant development and the neurogenetic disorder for which they were diagnosed. Phase 2 includes direct parent coaching around parent-child interaction based on an empirically based parent-mediated early intervention and repeated comprehensive assessments of family and child functioning.

2. Project Personnel

1. Will this project be led by a STUDENT (undergraduate, graduate) or TRAINEE (resident, fellow, postdoc), working in fulfillment of requirements for a University course, program or fellowship?

   No

2. List all project personnel beginning with principal investigator, followed by faculty advisor, co-investigators, study coordinators, and anyone else who has contact with subjects or identifiable data from subjects.

   - List ONLY those personnel for whom this IRB will be responsible; do NOT include collaborators who will remain under the oversight of another IRB **for this study**.
   - If this is Community Based Participatory Research (CBPR) or you are otherwise working with community partners (who are not functioning as researchers), you may not be required to list them here as project personnel; consult with your IRB.
   - If your extended research team includes multiple individuals with limited roles, you may not be required to list them here as project personnel; consult with your IRB.

   The table below will access campus directory information; if you do not find your name, your directory listing may need to be updated.
   If a change to the Principal Investigator is requested during the course of the study, a PI Change Form must be submitted.

Liaison	Last Name	First Name	Department Name	Role	
University of North Carolina at Chapel Hill (UNC-CH)					
	Hazlett	Heather	Psychiatry - General	Principal Investigator	view
	Wheeler	Anne	Psychiatry - Research	Co-investigator	view
	Okoniewski	Casey	Psychiatry - Carolina Institute for Developmental Disabilities	Study Coordinator	view
★	Edwards	Anne	Carolina Institute for Developmental Disabilities	Research Assistant	view
	Grzadzinski	Rebecca	Psychiatry - Carolina Institute for Developmental Disabilities	Research Assistant	view
	McNeilly	Heidi	Carolina Institute for Developmental Disabilities	Research Assistant	view
	Prince	Emily	Carolina Institute for Developmental Disabilities	Research Assistant	view

External Institutions

Liaison	Last Name	First Name	Department Name	Role	
Children's Hospital Colorado					
★	Villagomez	Adrienne		Other	view

Research Triangle Institute (RTI International)				
	Wheeler	Anne	External Site PI	view
	Raspa	Melissa	Co-investigator	view
	Turner-Brown	Lauren	Co-investigator	view
⭐	Okoniewski	Casey	Study Coordinator	view
	Andrews	Sara	Research Assistant	view
	Daas	Manisha	Research Assistant	view
	Edwards	Anne	Research Assistant	view
	Scott	Samantha	Research Assistant	view

If your research includes personnel from a UNC Health Network Entity (NE), the UNC Health Office of Research Support and Compliance (ORSC) will review your IRB application and/or submitted UNC Health Collaboration Survey. You may be contacted by ORSC for additional information. IMPORTANT: In addition to obtaining IRB approval, you must also receive ORSC clearance for project personnel employed by the NE site(s). Project personnel MAY NOT proceed with research activities until you have obtained both approval from the IRB and clearance from the NE. Upon completed ORSC review, an ORSC NE Clearance Form will be provided and uploaded to the IRB application Study Documents section.

NOTE: The IRB database will link automatically to UNC Human Research Ethics Training database and the UNC Conflict of Interest (COI) database. Once the study is certified by the PI, all personnel listed (for whom we have email addresses) will receive separate instructions about COI disclosures. The IRB will communicate with the personnel listed above or the PI if further documentation is required.

3. If this research is based in a center, institute, or department (Administering Department) other than the one listed above for the PI, select here. Be aware that if you do not enter anything here, the PI's home department will be AUTOMATICALLY inserted when you save this page.

Department	Psychiatry - Carolina Institute for Developmental Disabilities

3. Funding Sources

1. Is this project funded (or proposed to be funded) by a contract or grant from an organization EXTERNAL to UNC-Chapel Hill?

   Yes

Is UNC-CH the **direct** recipient of any Federal funding for this study? You should answer 'yes' *only* if you are the grantee. You should answer 'no' if you are the recipient of a sub-award or contractor under the grant.

No

Funding Source(s) and/or Sponsor(s): Please list all entities that are providing monetary support or supplies (e.g., study drug, gifts, devices at no cost, or others that provide in-kind services).

Sponsor Name	UNC Ramses Number	Sponsor Type	Prime Sponsor Name	Prime Sponsor Type	Sponsor/Grant Number	Detail
John Merck Fund	Currently Not Available	Foundation				view

2. Is this study funded by UNC-CH (e.g., department funds, internal pilot grants, trust accounts)?

   No

3. Is this research classified (e.g. requires governmental security clearance)?

   No

4. Is there a master protocol, grant application, or other proposal supporting this submission (check all that apply)?

✔	Grant Application
✘	Industry/Federal Sponsor Master Protocol
✘	Student Dissertation or Thesis Proposal
✘	Investigator Initiated Master Protocol
✘	Other Study Protocol

5. Is this a Clinical Study?
Check YES if this study involves research using human volunteers that is intended to add to medical knowledge. There are two main types of clinical studies: clinical trials and observational studies. Do NOT check yes merely because you are conducting research in a clinical setting or using clinical data.
Click here for additional definition of "Clinical Study"
Yes

Does your study ONLY include observational activities?
Observational study: A type of clinical study in which participants are identified as belonging to study groups and are assessed for biomedical or health outcomes. Participants may receive diagnostic, therapeutic, or other types of interventions, but the investigator does not assign participants to a specific interventions/treatment. A patient registry is a type of observational study.
Yes

Will this clinical trial be listed in ClinicalTrials.gov, either by you or the sponsor?
Click here for ClinicalTrials.gov Guidance Information
Yes

Choose the appropriate Phase designation for this clinical trial.

✔	Pilot Study
✘	Phase I
✘	Phase I/II
✘	Phase II
✘	Phase III
✘	Phase IV
✘	Other

4. Screening Questions

The following questions will help you determine if your project will require IRB review and approval.

The first question is whether this is RESEARCH (click for details)

1. Does your project involve a systematic investigation, including research development, testing and evaluation, which is designed to develop or contribute to generalizable knowledge? PLEASE NOTE: You should only answer yes if your activity meets all the above.
Yes

The next questions will determine if there are HUMAN SUBJECTS (click for details)

2. Will you be obtaining information or biospecimens through intervention or interaction with the individual, and use, study, or analysis of the information or biospecimens? This would include any communication or interpersonal contact between investigator and subject such as using in-person or online questionnaires/surveys, interviews, focus groups, observations, treatment interventions, etc. PLEASE NOTE: Merely obtaining information FROM an individual does not mean you should answer 'Yes,' unless the information is also ABOUT them.
Yes

3. Will you be obtaining, using, studying, analyzing, or generating identifiable private information or identifiable biospecimens collected through means other than direct interaction? This would include data, records or biological specimens that are currently existing or will be collected in the future for purposes other than this proposed research (e.g., medical records, ongoing collection of specimens for a tissue repository).
OR
Will you be using human specimens that are not individually identifiable for FDA-regulated in vitro diagnostic (IVD) device investigations?
Yes

The following questions will help build the remainder of your application.

4. Will subjects be studied in the Clinical and Translational Research Center (CTRC, previously known as the GCRC) or is the CTRC involved in any other way with the study? (If yes, this application will be reviewed by the CTRC and additional data will be collected.)

No

5. Does this study directly recruit participants through the UNC Health Care clinical settings for cancer patients **or** does this study have a focus on cancer or a focus on a risk factor for cancer (e.g. increased physical activity to reduce colon cancer incidence) **or** does this study receive funding from a cancer agency, foundation, or other cancer related group? (If yes, this application may require additional review by the Oncology Protocol Review Committee.)

No

6. Is the UNC Chapel Hill IRB taking or being asked to take responsibility for the oversight of research by individuals, groups or organizations outside of UNC Chapel Hill? Or you are asking the UNC Chapel Hill IRB to cede review to an External IRB. If so, a reliance agreement will need to be executed prior to conducting any research activities. See guidance.

Yes

5. Multi-site Study Information

1. Is UNC-CH the Lead Site or Coordinating Center or Sponsor of a multicenter project?

Yes

Lead Site/Coordinating Center addendum

The Lead Site/Coordinating Center addendum is not required if you are relying on an external IRB. In the attachments section, click Lead Site/Coordinating Center addendum and select the Not Yet Available / Not Applicable checkbox.

2. Is UNC-CH taking or being asked to take responsibility for the oversight of research by individuals, groups or organizations outside of UNC-CH?

Yes

When a collaborator(s) outside of UNC-CH is (a) exercising authority or responsibility on behalf of a group or organization, (b) performing activities designated by a group or organization, or (c) using the collaboration for scholarly advancement (e.g., promotion, tenure) at a group or organization, complete the following information:

External Institution	Has or will the external institution agree to rely on the UNC-Chapel Hill IRB?	Local Consent Forms and Ads	Local Context Worksheet	Agreement
Children's Hospital Colorado	Yes			
**Personnel**	**Role**	**Ethics**	**CV**	**MD License**
Adrienne Villagomez	Other			

View sIRB Attachments

External Institution	Has or will the external institution agree to rely on the UNC-Chapel Hill IRB?	Local Consent Forms and Ads	Local Context Worksheet	Agreement
Research Triangle Institute (RTI International)	Yes			
**Personnel**	**Role**	**Ethics**	**CV**	**MD License**
Sara Andrews	Research Assistant			
Manisha Daas	Research Assistant			
Anne Edwards	Research Assistant			
Casey Okoniewski	Study Coordinator			
Melissa Raspa	Co-investigator			
Samantha Scott	Research Assistant			

Lauren Turner-Brown	Co-investigator			
Anne Wheeler	External Site PI			

View sIRB Attachments

Researchers are reminded that additional approvals may be needed from relevant "gatekeepers" to access subject.

Location

1. Are UNC-affiliated researchers involved in research conducted at any locations outside of the United States?

No

Part A. Questions Common to All Studies

A.1. Background and Rationale

A.1.1. Provide a summary of the background and rationale for this study (i.e., why is the study needed?). If a complete background and literature review are in an accompanying grant application or other type of proposal, only provide a brief summary here. If there is no proposal, provide a more extensive background and literature review, including references.

Fragile X syndrome (FXS) is the leading known single-gene cause of autism spectrum disorder (ASD) and the most common inherited form of intellectual disability, resulting in significant functional impairments (Raspa, Wheeler, & Riley, 2017). The mutation of the FMR1 gene has broad implications for the family. Expansions of the gene occur over generations; therefore, identification of one individual with FXS results in the identification of at least one, and often many, additional family members with an FMR1 mutation. Frequently, multiple children are born into a family before a diagnosis is made (Bailey, Raspa, Bishop, & Holiday, 2009), which leads to broad family consequences (Raspa, Bailey, Bann, & Bishop, 2014). Despite documentation that symptoms emerge in the first year of life (Burris, Barry-Anwar, Sims, Hagerman, Tassone, & Rivera, 2017; Roberts et al., 2009; Swanson et al., 2018), the average age of diagnosis is 3 years of age for males with FXS (Bailey et al., 2009). Females with FXS and individuals with the premutation are often diagnosed even later as they typically have a less severe phenotype. The delay of diagnosis can reduce timely access to interventions that could have significant consequences for the developmental trajectories of these children. Furthermore, the delay in diagnosis has significantly limited the ability of researchers to understand the natural progression of symptomology and thereby better address treatment options.

Recent initiatives to identify FXS earlier include a national discussion about newborn screening for FXS (Bailey, Skinner, Davis, Whitmarsh, & Powell, 2008; Riley & Wheeler, 2017). Although FXS does not currently meet criteria for a traditional newborn screening panel (NBS), FX is an ideal condition to explore voluntary expanded NBS procedures because of the lack of obvious features at birth, severity, and impact on the broader family system. It is necessary to identify the benefits of early identification to determine whether NBS for FX would be of true benefit to the individual, family, society, and public health support systems.

The rationale for including infants with FXS in this intervention study extends to infants identified with other rare neurogenetic disorders currently not included on traditional newborn screening panels (NBS). The delay in diagnosis has significantly limited the ability of researchers to understand the natural progression of symptomology and thereby better address treatment options. Early access to effective interventions could have significant consequences for the developmental trajectories of these children. In addition, identifying the benefits of early intervention will determine whether NBS for these disorders would be of true benefit to the individual, family, society, and public health support systems.

A.1.2. State the research question(s) (i.e., specific study aims and/or hypotheses).

The primary goal of the proposed project is to develop and test, through an iterative process, an intervention to address and support the development of infants with a confirmed diagnosis of neurogenetic disorders that include a risk for developmental delay or intellectual and developmental disabilities identified at birth through the Early Check program (IRB #18-0009) or eligible self-referrals. PIXI aims to utilize the foundational knowledge available around the development of and early intervention for at-risk infants to both understand the needs of and provide intervention services for families of infants diagnosed presymptomatically with these disorders. We aim 1) develop PIXI with a pilot sample of families, 2) test the preliminary effects of PIXI on infant and parent outcomes. Specific infant outcomes include communication, motor, cognitive skills and

autism spectrum disorder symptoms. Parental outcomes include parenting efficacy.

A.2. Subjects

A.2.1. Total number of subjects proposed across all sites by all investigators (provide exact number; if unlimited, enter 9999):

120

A.2.2. Total number of subjects to be studied by investigators being provided oversight by the UNC IRB. (provide exact number; if unlimited, enter 9999):

120

A.2.3. If the above numbers include multiple groups, cohorts, or ranges or are dependent on unknown factors, or need any explanation, describe here:

The 120 participants above will typically be made up of parent-infant dyads (one parent and infant). However, if a second parent asks to participate, they may be enrolled as well and will be asked to participate in the same activities as the first parent.

A.2.4. Do you plan to enroll subjects from these vulnerable or select populations:
If you will include children, prisoners or nonviable neonates or neonates of uncertain viability, please check the appropriate category below and complete the additional sections.

You should check "Pregnant women" if you specifically intend to recruit women who are pregnant or are not excluding pregnant women in biomedical research that is greater than minimal risk. Do not check if you are conducting a survey of the general public or conducting secondary data analysis or chart review not aimed at pregnant women.

Only check UNC-CH Student athletes, athletic teams, or coaches if you have specific plans to enroll these subjects. This is not applicable for intramural or club sports. For definitions and guidance see SOP 1201: Vulnerable subjects in research.

✔ Children (under the age of majority for their location)

Any minor subject who attains the age of majority during the course of the research study must provide consent as an adult, unless consent has been waived, which is requested in section D.3.1.

✘ Pregnant women

✘ Nonviable neonates or neonates of uncertain viability

✘ Prisoners, others involuntarily detained or incarcerated (this includes parolees held in treatment centers as a condition of their parole)

If an enrolled participant becomes incarcerated during the course of the research, they must be removed from the research project until such time as the IRB (and OHRP for NIH funded projects) approves the study to include prisoners, unless there is an immediate risk to the participant from ending treatments under the protocol.

✘ UNC-CH Student athletes, athletic teams, or coaches

A.2.5. Based on your recruitment plan and target sample population, are you likely to include any of the following as subjects? Select all that apply. This is not applicable to secondary data analysis or chart review.

Based on your responses, the consent form builder will insert the required text into your consent form template.

✘ Decisionally impaired individuals

(e.g., Mini mental state examination (MMSE), Montreal cognitive assessment (MOCA))

✘ Children who are wards of the State (Foster children)

✘ Non-English-speaking individuals

✘ UNC-CH Students

✘ UNC-CH Employees

✖ People, including children, who are likely to be involved in abusive relationships, either as perpetrator or victim.
This would include studies that might uncover or expose child, elder or domestic abuse/neglect. (See SOP Appendix A)

A.2.6. If any of the above populations are checked (excluding 'Decisionally impaired individuals' and 'Children who are wards of the State (Foster children)'), please describe your plans to provide additional protections for these subjects.

No Answer Provided

A.2.7. Age range of subjects:

Minimum age of subject enrolled	0
	months
Maximum age of subject enrolled	99
» If no maximum age limit, indicate 99	
	years

A.2.A. Children

Research involving children (45 CFR 46 Subpart D or 21 CFR 50 Subpart D)

A.2.A.1. Why is it necessary to involve children as subjects for this research? If the study addresses a condition that particularly affects children, please explain.

This study aims to develop and test an intervention to address and support the parent understanding and development of infants with a confirmed diagnosis of a rare neurogenetic disorder. In North Carolina, identification of these infants at birth will provide families with access to a family coordinator through the Early Intervention program who will connect with the family on a regular basis and make referrals to specific providers (e.g., physical therapist, speech/language pathologist) when the child begins to show symptoms warranting those services. Although earlier access to these services may in itself make a difference for these children, the timing, quality, and dosage of these services will differ across states and will depend on the emergence of symptoms. It is hypothesized that the greatest impact will come from a more intensive intervention that is implemented pre-symptomatically or when the very first symptoms are observed. To fully understand the possible preventative effects of early identification and intervention during infancy on symptomatology, it is necessary to include children in this study.

A.2.A.2. Describe potential for direct benefit to children participating in this study OR if no prospect of direct benefit to children participating in this study, explain how research is likely to yield generalizable information about the condition. If applicable, please explain how benefit would differ for children randomized to active (i.e. treatment or intervention) versus placebo (i.e inactive or control) groups.

Infants involved in the study will receive intervention that addresses the core deficits seen in children with neurogenetic disorders (communication, social, sensory, cognition, motor skills) which may benefit them in the future progression of the condition. Additionally, children will gain access to early intervention pre-symptomatically or as first symptoms emerge, which may be associated with improved developmental outcomes. The intervention will include parent education about the disorder as well as direct parent coaching around parent-child interactions and repeated comprehensive assessments of family and child functioning. This parent-mediated approach aims to achieve two goals (1) general enrichment of the core interactive social experience for infants and (2) specific attention within this to addressing any emerging atypical behaviors that might be expected at this age and their interactional consequence.

A.2.A.3. Describe the unique risks associated with children AND discuss your plans to minimize the risks and provide additional protections.

To minimize the risk of an infant becoming stressed, all assessment and intervention sessions will be conducted in a setting comfortable to the child. Intervention sessions will be conducted in the home. Assessments will be conducted in home or in a laboratory setting that will be designed to be infant friendly, with appropriate furniture and toys. Parents of the infants will present during the assessments and infants will have access to them if they become distressed.

If an infant presents an unusually fearful reaction and cannot be easily soothed or redirected, as decided by the interventionist/assessor or the parent, during the developmental assessments or the home intervention sessions the task will be discontinued and resumed only when the infant's behavior indicates an ability to participate without significant fear or frustration. If the infant seems unable to participate due to fear or frustration or the interventionist/assessor or the parent feels the task will be too stressful for the child, the assessment/intervention will be discontinued.

Some children may not like the developmental assessments. The risk for discomfort with the developmental assessment is no more than they would experience as part of their routine developmental evaluations. During remote developmental assessment administration parents will have trained licensed psychologists or research assistants with extensive experience testing young children with and without disabilities immediately available for support.

Infants may be uncomfortable while wearing the LENA clothing. The LENA has been used with thousands of children with very few reports of problems, therefore we do not anticipate this being an issue.

There is a slight risk that the heart rate monitor will be uncomfortable to the infants. Researchers will be well trained to help guide parents in needed adjustments to improve comfort. Caregivers will also be assured that they can terminate any portion of the study, including collection of heart rate, at any time.

A.3. Inclusion/exclusion criteria

A.3.1. List required characteristics of potential subjects (i.e., inclusion and exclusion criteria). If not covered, list also characteristics that would preclude their involvement.

All birthing mothers in North Carolina will be invited to participate in Early Check (IRB #18-0009), a voluntary research program where newborns will be screened for a carefully selected panel of conditions. Early Check is a complement to the state-mandated newborn screening and is conducted in collaboration with the North Carolina State Laboratory of Public Health, utilizing existing newborn blood spots to test for these conditions. It will be offered under a research protocol with parental permission. Children who receive a diagnosis after participation in the Early Check Screening and speak English will be eligible for the intervention and invited to participate.

Self-referrals with infants 12 months of age or younger who have received a diagnosis which was not sought solely due to parental concerns about the infant and who speak English will also be eligible. Infants may not be blind or have a severe hearing impairment as the intervention and assessments are not appropriate for these children. Infants who receive a diagnosis of FXPM after participation in Early Check, speak English, and show signs of delay will be given information about the study and added to the wait-list if interested. FXPM infants who don't show signs of delay but may have increased genetic risk or their parents show anxiety regarding their child's development will also be given information about the study and added to the wait-list if interested. Infants will include both sexes. Families of any race/ethnicity will be invited to participate.

A.3.2. Justify any exclusion based on race, gender or ethnicity

Participants will not be excluded based on race, gender, ethnicity. However, English must be the primary language spoken in the home because all assessment measures and intervention protocol are in English.

A.3.3. Will pregnant women or women who become pregnant be excluded?

No

A.4. Study design, methods and procedures

Your response to the next question will help determine what further questions you will be asked in the following sections.

A.4.1. Will you be using any **methods or procedures commonly used in biomedical or clinical research** (this would include but not be limited to drawing blood, performing lab tests or biological monitoring, conducting physical exams, administering drugs, or conducting a clinical trial)?

Yes

A.4.2. Describe the study design. List and describe study procedures, including a sequential description of what subjects will be asked to do, when relevant.

This study will include an open trial of PIXI with a pre-post design. Below is a timeline for participants

from pre-enrollment through study completion.

Overall sequence for participants:

1. Parents enroll in Early Check to permit newborn screening. Parents of infants who screen positive will be contacted and asked to permit confirmatory testing. When confirmatory testing confirms diagnosis, families will attend a genetic counseling session virtually or at the UNC Carolina Institute of Developmental Disabilities (CIDD) to receive this diagnosis and accompanying genetic counseling when the infant is approximately 2-3 months of age.
2. At this Genetic counseling visit, families will be informed about associated studies, including the present PIXI study. Parents who express interest in this study can enroll during this genetic counseling visit or take-home information about PIXI and contact the study team to enroll anytime during the infant's first 12 months of life. Families that wish to further contemplate enrollment in PIXI after their genetic counseling appointment will be called a week after their appointment to assess any further interest in the study. Parents of infants with FXPM diagnosed through Early Check will be invited to participate in PiXI when there are available slots. Eligible families will be provided with information about PIXI and if interested, added to the wait-list. Additionally, families with rare neurogenetic disorders who have not been identified through Early Check may self-refer to the study or be referred by a provider. These families who contact the study team and express interest in the study, will be provided the informational handout (attached). If families wish to enroll, they will be added to the waitlist and, when applicable, will schedule an appointment for their Baseline/Time 1 assessments. After enrollment in the study, the procedures for self-referrals and those identified through Early Check will be identical.
3. Informed consent will be obtained from families once they express interest in enrollment. Families may review the consent in person or via phone or video-chat if a member of the research team is unable to schedule an in-person visit based on the distance of the family home; written consent will be obtained from all participants. Enrollment in PiXI will require families to permit sharing of relevant data from Early Check (e.g., confirmation of diagnosis, demographics, infant and family assessments conducted during genetic counseling visit). Non-Early Check families will be asked to provide confirmation of the diagnosis.This data will count as part of the baseline assessment data for PIXI. Additionally, if the family has recently completed relevant assessments with an outside provider or through a separate research study, families may share these assessment results with study team members rather than complete the assessment again. If participants are unable to provide all necessary time 1 assessments, they will be asked to complete them before beginning Phase 1 of the intervention. Participants will be asked to sign an authorization to disclose information form, allowing the study team to obtain assessments from the outside provider or study (see attached form). The consent process is further detailed in section D.1.1.
4. Baseline developmental assessments (time 1 assessments) will be conducted as part of Early Check (IRB #18-0009) and shared with the present study. For families not enrolled in Early Check, baseline assessments will be scheduled by the team or sharing of relevant information will be coordinated prior to beginning Phase 1. Assessments are estimated to take around 90 minutes for the child portion (depending on the age of the child—older children will take longer), and parent rating-scales will take approximately 2 hours or less to complete. Some parent report assessments may be completed via electronic questionnaire.
5. PIXI study staff will be given the names and contact information of interested families and will contact those families within 1 week of the EC genetic counseling visit or baseline assessment to schedule intervention sessions. Phase 1 of PIXI will then commence. Phase 1 will occur within the first 3-6 months of enrollment. Phase 1 involves approximately 4-12 parent video calls through a secure video calling system, i.e. Zoom or other video-conferencing service. Families will be provided a device capable of video calling (if needed), upon receiving the device they will be asked to sign an agreement which states they understand any purchases made are their own responsibility and that the device is to be returned at the conclusion of the intervention (see attached). Some of the sessions may be scheduled as an in-home visit depending on the availability of the interventionist and family and family need. In addition, Phase 1 therapists will coordinate with family's existing early intervention provider (if parents grant permission; see attached authorization form) to ensure these providers are aware of the diagnosis and provide information about the disorder to those providers. Session topics will be flexible to provide the opportunity to address parent's immediate needs and concerns, provide psychoeducation, and build rapport in preparation for Phase 2 of the intervention. Each session will include the following: (1) needs assessments targeted at collecting information regarding parent concerns and family priorities, obtained through structured interviewing and questionnaires, (2) implementation of parent coaching techniques (Rush & Sheldon, 2013), (3) feasibility testing of early intervention programming targeting known

delays in this population, (4) routines focus, and (5) psychoeducation and resource provision (if applicable) around area of concern. Phase 1 will last for approximately 3 months. After the first introduction session, each session will include a 15-minute parent check in, 30 minutes of session topic discussion, and 15 minutes of intervention techniques and wrap up. Video calls will be audio recorded and coded to record parent reports of developmental changes over time.

6. Post implementation of Phase 1 of PiXI, time 2 assessments will be conducted. Assessments are estimated to take up to 2 hours for the child portion (depending on the age of the child—older children will take longer), and parent rating-scales will take up to 2 hours to complete. Some parent report assessments may be completed via electronic questionnaire. If families are unable to attend a full assessment session, sessions may be broken up over several days to accommodate need. Parents may complete self-report questionnaires and send them back to study team. If families will be receiving or completing relevant assessments from an outside provider or study, they may opt to share those results with the study team rather than complete the assessment again. A study member unknown to the family will complete social validity and post-intervention interview with the family on the phone, over a video-call, or in person.
7. Families will receive Phase 2 of PIXI following their Time 2 assessment. Initially, Phase 2 followed the British Autism Study of Infant Siblings- Video Feedback Intervention to Promote Positive Parenting (iBASIS-VIPP; Juffer, Bakermans-Kranenburg, & Van IJzendoorn, 2008, Green et al., 2015). Upon review of the first four participants, interventionists felt that the iBASIS-VIPP parent coaching methodology was not effective with this specific population, therefore we propose to use a modified version of Infant Start (Rogers et al. 2014), which utilizes a more concrete and directive coaching methodology. We will work with one of the creators of Infant Start, Dr. Laurie Vismara to modify the intervention to meet the needs of families with FX. Dr. Vismara will support research team in the training, fidelity, and implementation of the intervention through video observation. Dr. Vismara will receive no identifiable information other than information which may be disclosed within the video. See section A.11 and the attached contract for further details on her role as an educational consultant and confidentiality agreements. The infant enrolled who is still participating will complete iBASIS VIPP and all future infants enrolled will participate in Infant Start; results will be compared at the end of the intervention. Phase 2 will consist of approximately 6 to 20 sessions occurring on a weekly or biweekly basis. Number of sessions will be based on family availability and need. Sessions will be conducted by a trained interventionist and will occur via telehealth. Some sessions may be conducted in person based on family need. Intervention will target symptoms related to developmental delay and ASD symptomology. The first session of phase 2 will be used to develop treatment goals; interventionist will complete an informal play assessment with parent and child and fill out the Early Start Denver Model Checklist which allows interventionist to inventory child's developmental skills. Interventionist will coach parents in utilization of intervention strategies to meet developmental goals using a variety of everyday routines and play opportunities. Interventionist and family will have opportunity to discuss and review strategies. Interventionist will video record sessions. Video recordings will later be coded for parent responsivity and fidelity. The previous i-BASIS-VIPP program consisted of a combination of up to 12 in-home (scheduled as needed) and telehealth sessions over a period of 5 months. The 10 sessions included (1) a preliminary session to establish goals with the parent, (2) six intervention sessions (delivered weekly to bimonthly) with a theme building on strategies learned and practiced from the previous session, and (3) up to five booster sessions to consolidate learning. In the six intervention sessions, the first two focus on infant behavior (with parent behavior alluded to indirectly), the second two address parent behavior, and the final two sessions examine more complex chains of interaction. Interventionist video recorded parent-child interactions. Video recordings will later be coded for parent responsivity.
8. After completing Phase 2 of the intervention, time 3 assessments will be conducted. Assessments are estimated to take up to 2 hours for the child portion (depending on the age of the child—older children will take longer), and parent rating-scales will take up to 2 hours to complete. If families are unable to attend a full assessment session, sessions may be broken up over several days to accommodate need. Parents may complete self-report questionnaires and send them back to study team. Some parent report assessments may be completed via electronic questionnaire. If families will be receiving or completing relevant assessments from an outside provider or study, they may opt to share those results with the study team rather then, complete the assessment again. A study member unknown to the family will complete social validity and post-intervention interview with the family on the phone, over a video-call, or in person.
9. After completion of the intervention, four follow-up assessments will be completed. Time 4 assessments will be completed at approximately 18 months. Time 5 assessments will be completed at approximately 24 months. Time 6 assessments will be completed at approximately 30 months. Time 7 assessments will be completed at approximately 36 months. Assessments will not occur past 36 months therefore

participants that enroll late may not complete all assessments (i.e. if a participant completes their post-phase 2 assessment at 18 months, they would only complete 2 follow up assessments). For children enrolling at older ages, the combination of measures administered may differ slightly at different timepoints to accommodate the age and/or developmental level of the child.

Assessments:

A member of the research team will contact each family to schedule an assessment date. All in person assessments will occur in our assessment lab in the UNC School of Medicine Carolina Institute for Developmental Disabilities (CIDD), a regional UNC TEACCH Autism Program center, or in family homes. Additionally, assessments may occur in an alternative private location that is convenient to both the participant and the researchers or virtually if in-person assessments are not possible due to distance or availability. If families are unable to attend a full assessment session, sessions may be broken up over several days to accommodate need. Parents may complete self-report questionnaires and send them back to study team. Some parent report assessments may be completed via electronic questionnaire. Research staff will assess general development, motor skills, communication skills, autism symptoms, sensory concerns, infant engagement, eye-tracking visual attention, parental measures, and acceptability and satisfaction.

Table of Measures to be Completed at Each Assessment Time point:

Time 1

Time 2

Time 3

Time 4

Time 5

Time 6

Time 7

Direct Child Assessment

Developmental Assessment of Young Children, 2nd Edition (DAYC-2) (remote assessment)

x

x

x

x

x

x

x

Parent-Administered Neurodevelopmental Assessment (PANDABox) (remote assessment)

x

x

x

x

x

x

x

TELE_ASD_PEDS (remote assessment)

x

x

x

x

Bayley Scales of Infant and Toddler Development (Bayley)

x

x

x

x

x

x

x

Peabody Developmental Motor Scales, Second Edition (PDMS-2)

x

x

x

x

x

x

x

Autism Observational Scale for Infants (AOSI)

x

x

x

Autism Diagnostic Observation Schedule, Second Edition (ADOS-2)

x

x

Adamson Engagement States

x

x

x

Eye-tracking tasks

x

x

x

Parent Report: Child

Current Concerns & Interventions

x

x

x

x

x

x

x

Feeding/NeoEat/PediEat

x

x

x

x

x

X

x

Brief Infant Sleep Questionnaire (BISQ Adapted)

x

x

x

x

x

X

x

Vineland Adaptive Behavior Scales, Third Edition (Vineland-3)

x

x

x

x

x

X

x

Infant Behavior Questionnaire (IBQ)/Children's Behavior Questionnaire (CBQ)

x

x

x

x

x

X

x

Communication and Symbolic Behavior Scales Developmental Profile (CSBS-DP)

x

x

x

x

X

x

Modified Checklist for Autism in Toddlers (MCHAT)

x

x

Sensory Profile, Second Edition (SP-2)

x

x

x

x

x

x

x

Repetitive Behavior Scale

x

x

x

x

x

Parent Report: Self

Parent Engagement Intervention

x

x

Demographics

x

x

x

x

x

x

x

Edinburgh Postnatal Depression Scale (Edinburgh)

x

x

x

Patient Health Questionnaire-9 (PHQ-9)

x

x

x

x

Behavior Rating Inventory of Executive Functioning, Second Edition (BRIEF2A)

x

State-Trait Anxiety Inventory (STAI)

x

x

x

x

x

x

Parenting Stress Index, Fourth Edition (PSI-4: SF)

x

x

x

x

x

x

Medical Outcomes Study-Social Support Scale (MOS-SSS)

x

x

x

x

x

X

Social Validity & Parent Interview

x

x

Direct Administration Measures:

The first three direct measures, the PANDABox and the TELE-ASD-PEDS are remote measures which will replace the in-person battery if needed and the assessment is conducted remotely.

The Developmental Assessment of Young Children, 2nd Edition (DAYC-2; Voress, Maddox & Hammill, 2012) is a standardized assessment that utilizes observation, interview, and direct assessment to examine early developmental skills in the areas of cognition, fine motor, gross motor, expressive language, and receptive language. The PIXI study will be using an adapted version for telehealth administration that takes approximately 20-30 minutes for administration.

Parent-Administered Neurodevelopmental Assessment (PANDABox; Kelleher et al., 2020) To be administered at every timepoint if remote.

NOTE: These procedures have previously been approved by the UNC IRB (#18-1778).

The RDA utilizes behavioral and spectral measures of attention, temperament, language, play, and social presses. The full battery will take approximately 30-40 minutes to complete. Team members will be provided direct training on the RDA.

A week before the scheduled assessments parents will be sent a demonstration video along with the assessment kit (programmed laptop, HD webcam, heart monitor, phone headset, Language ENhancement Analysis (LENA) kit and vest, play mat, and toys).

The day before the session, parents will record a sample of infant vocalizations using the LENA system. LENA uses a non-invasive wearable sensor that records language input and output and accompanying acoustic information, providing norm-based data on adult/child word count and conversational turn taking. The morning of the recording the examiner will contact the parent to remind them to turn on the LENA, which will continuously record for 16 hours. The examiner will also call the family the night before to ensure all systems are working correctly.

The following day, the examiner and parent will communicate via phone and screen share facilitated by Bomgar software. The examiner will call the parent, walk them through opening Bomgar and remotely access the loaned computer. A trained RA/Examiner will be with the parent throughout the whole assessment via videoconferencing and on the phone guiding the parent through the developmental assessment tasks. By remoting in, the RA/Examiner will have full control over the parent's screen to help troubleshoot any difficulties. Additionally, each step of the assessment activities will be described in detail by the RA/Examiner with accompanying PowerPoint slides to provide visual and written instruction as well.

Once accessed, the heart monitor and video software will be launched. During all tasks the infant will wear a heart rate monitor, as atypical heart activity has previously been observed in infants with developmental delays. Throughout assessment activities, infants will wear a small transmitter that will be placed on their chest using standard electrodes (similar to a Band Aid). Devices we will use include CamNTech Actiheart Cardiac Monitors. This monitor is approved by the FDA. The examiner will coach the parent to start the monitor, prepare the electrodes, and place them on the infant's chest. They will be presented with a picture as to where to place the electrode. These activities have been implemented with over 100 families in Dr. Kelleher's lab, with little to no difficulty expressed by parents or discomfort observed with the infant.

Once all technical systems are in place, the following tasks will be administered:

- Visual Attention Task: Looking (eye gaze) will be recorded by an HD webcam while infants view a three-minute children's video on the loaned laptop. The parent will provide up to three prompts to engage the child if they look away for >5 s. The task provides direct measures of looking time, sustained attention, and heart rate variability.
- Developmental/Free Play Assessment: The examiner will cue the parent to introduce play materials without prompting or modeling. Video coding of the task will provide measures of play diversity, stereotypies, parent-child engagement, sustained attention, and heart rate variability during play.
- Temperament Assessment: During an arm restraint task (to probe for negative affect), the parent will

give the child an engaging toy for 30s, gently restrain their arms to prevent engagement for 30s, then return the toy and say, “It’s okay [name], you can have it. [Parent] was just teasing.” During a bubbles task (to probe for positive affect), the parent will blow bubbles while reading 5 standard prompts. Coding of video will provide measures of positive affect (e.g. smiling), negative affect (e.g. escape behaviors), and heart rate modulation.

- Story Time: The examiner will cue the parent to read a book with their infant just as they would at home.
- Parent-Child Interaction: The examiner will let the parent and child freely interact for a 10-minute episode of videotaped, unstructured toy play. The dyad will sit on a floor mat with a small set of toys. The parent will be instructed to engage in play with the child as they typically would at home, with or without the toys.

Once the assessment is complete, the examiner will review with the parents how to re-pack the return shipping box for the materials. All assessment materials will be returned either via pre-paid box that can be scheduled for pickup at the family’s convenience, or dropped off at a designated location, whichever the family prefers. The kits are returned to the team after each visit.

TELE-ASD-PEDS: A Telemedicine based ASD Evaluation Tool for Toddlers and Young Children. The TELE-ASD-PEDS was developed by researchers at Vanderbilt University to assess remotely autism symptomology. The TELE-ASD-PEDS will be administered during remote visits at 24- and 36-months, or, if the parent reported M-CHAT score is elevated and indicative of potential ASD symptomology, at 18 or 30 months. If the infant has received a diagnosis of autism prior to any of these timepoints, the protocol will not be completed.

Bayley Scales of Infant and Toddler Development-III (Bayley, 2006). The Bayley-III is a standardized measurement of early developmental skills across the areas of cognition, language, motor, and social-emotional abilities for infants 1-42 months.

Peabody Developmental Motor Skills, 2nd edition (PDMS-2) (Folio &Fewell, 2000) This early childhood motor development program contains six subtests that assess the motor skills of children from birth through 5 years.

Autism Observation Scale for Infants (AOSI) (Bryson et al., 2007). The AOSI 18-item direct observational measure designed to detect and monitor putative signs of autism in infants aged 6–18 months.

Autism Diagnostic Observation Schedule-2 (ADOS) (Lord et al., 2012) ADOS-2 is a semistructured, standardized assessment of communication, social interaction, play, and restricted and repetitive behaviors.

Parent Report Measures:

Demographics, Current Concerns & Interventions. These study developed measures will gather demographic data from participants, along with areas of current concerns in development, feeding, sleeping, behavior and participation in intervention services

Vineland Adaptive Behavior Scales, 3rd edition (VABS-3) (Sparrow, Cicchetti, & Saulnier, 2016) The Vineland-3 is a standardized parent report measure of language, motor, social/leisure, and overall adaptive functioning. The Vineland-3 is a standardized measure of adaptive behavior--the things that people do to function in their everyday lives. Whereas ability measures focus on what the examinee can do in a testing situation, the Vineland-3 focuses on what he or she actually does in daily life. The Vineland-3 is a norm-based instrument and the examinee's adaptive functioning is compared to that of others his or her age. Scores provided include an Adaptive Behavior Composite (Overall Score) and specific Domain and Subdomain scores (Communication [expressive, receptive written], Daily Living [community, domestic, personal], Socialization [play and leisure, interpersonal, coping skills], Motor [fine and gross]).

Infant Behavior Questionnaire Revised Very Short Form (IBQ-R VSF) (Rothbart & Gartstein, 2000) The Infant Behavior Questionnaire Revised-Very Short Form is a measure of infant temperament measuring positive affectivity, negative emotionality, and orienting and regulatory capacity.

Brief Infant Sleep Questionnaire (Sleep Questionnaire): The Sleep Questionnaire is an adaptive version of the Brief Infant Sleep Questionnaire (Sadeh, 2004). It includes 10 questions to gauge various aspects of sleep including duration, position, waking and difficulties.

*Repetitive Behavior Scale for Early Childhood (RBS-EC) (*Wolff et al., 2016). The RBS-EC is a questionnaire measure of restricted and repetitive behaviors designed for use in children from infancy through early school age. It is intended to capture individual differences across a broad range of behaviors associated with the

repetitive behavior domain.

Neonatal Eating Assessment Tool- Bottle feeding (NeoEAT- Bottle feeding) (Pados, Thoyre, Estrem, Park, & McComish, 2018) The NeoEAT – Bottle feeding is intended to assess observable symptoms of problematic feeding in infants less than 7 months old who are bottle feeding. The NeoEAT – Bottle feeding is intended to be completed by a caregiver that is familiar with the child's typical eating. This is most often a parent, but may be another primary care provider.

Neonatal Eating Assessment Tool- Breastfeeding (NeoEAT- Breastfeeding) (Pados, Thoyre, Estrem, Park, & McComish, 2018) The NeoEAT - Breastfeeding is intended to assess observable symptoms of problematic feeding in infants less than 7 months old who are breastfeeding. The NeoEAT – Breastfeeding is intended to be completed by a caregiver that is familiar with the child's typical eating. This is most often a parent, but may be another primary care provider.

Pediatrics Eating Assessment Tool (PediEAT) (Thoyre, Pados, Park, Estrem, McComish, & Hodges, 2018) The PediEAT is a parent-report measure developed to assess symptoms of feeding problems in children aged 6 months to 7 years.

Communication and Symbolic Behavior Scales (CSBS) (Wetherby & Prizant, 2002). This standardized measure is completed by parents to evaluate language predictors.

Modified Checklist for Autism in Toddlers (MCHAT) (Robins, Fein, & Barton, 2013) The Modified Checklist for Autism in Toddlers is a scientifically validated tool for screening children between 16 and 30 months of age that assesses risk for autism spectrum disorder (ASD).

Sensory Profile, 2nd edition (SP-2) (Dunn, 2014) Standardized forms completed by caregivers and teachers to assess children's sensory processing patterns.

Developmental Profile-4 (DP-4), Cognitive scale. Standardized forms completed by caregivers that assess cognitive skills. This measure will be used in place of the Bayley when in-person assessment is impossible.

*Behavior Rating Inventory of Executive Functioning, Second Edition, Adult Version (BRIEF-2A) (*Gioia et al., 2015) The BRIEF is a standardized measure that assesses executive and cognitive functioning. This measure will be added to obtain a general understanding of maternal skills.

Edinburgh Postnatal Depression Scale (Edinburgh; Cox et al., 1987). The Edinburgh is a ten-item checklist used to indicate if a parent is experiencing depressive symptoms. A score of 12 or more will trigger a referral for a mental health consultation and a suicide risk assessment if necessary.

Parent Health Questionnaire-9 (PHQ-9; Kroenke et al., 2001). The PHQ-9 screens for signs of depression and will help the team determine. A score of 10 or more on the PHQ-9 will trigger a referral for a mental health consultation and a suicide risk assessment if necessary.

Parenting Stress Index, Fourth Edition (PSI-4; Abidin, 2016). The PSI-4 is a standard measurement of stress in the parent-child relationship, identifying dysfunctional parenting and child adjustment problems.

Medical Outcome Study-Social Support Scale (MOS-SSS; Sherbourne & Stewart, 1991). The MOS-SSS provides an assessment of several domains of social support including tangible, emotional, affective, and positive support.

State Trait Anxiety Inventory (STAI; Spielberger, 1968). The STAI is a measurement of anxiety for adults that assists in distinguishing temporary and long-standing symptomology of anxiety and depression.

Post-Intervention Interview. A semi-structured interview will be completed with families after each phase of the intervention to facilitate ongoing refinement of the treatment manual. This interview will be audio recorded and will take approximately 20 minutes to complete. A full list of questions has been submitted.

Social Validity. A social validity measure will be developed to inquire about family satisfaction with aspects of the intervention including curriculum, timing, goals targeted, and perceived effects of the intervention. This measure will be completed by families after each intervention phase (e.g., at Time 2 and Time 3 assessments) and will take less than 10 minutes to complete.

*Parent Engagement Intervention (*PEI). To address barriers to session attendance and home practice, and to reduce the potential for differential attrition, we have adapted a brief motivational interviewing module for use at treatment outset and for reference during the intervention if needed. PEI is a manualized module shown to

improve treatment attendance and response in RCTs of parent-training programs. PEI is conducted over the phone and includes working individually with parents for 10 to 30 minutes to develop a collaborative plan to promote parent-identified goals by proactively addressing potential barriers to treatment attendance, persistence, and home practice.

Behavior Coding Measures:

Adamson Engagement States (Adamson, Bakeman, & Deckner, 2004) The engagement states coding system segments the child's activity into distinct and mutually exclusive periods. These periods characterize different ways the child might be engaged with objects, people, and/or symbols.

Eye-tracking tasks Our eye tracking battery will include the gap/overlap paradigm and a sequence learning task. The gap/overlap paradigm characterizes occulomotor and attentional function important for flexible and efficient information processing. A sequence-learning task designed by the National Children's Study Cognitive Health Team to asses early emerging cognitive flexibility will also be included. This addition of the sequence learning task will add minimal time commitment for the families, all task will take about 10-15 minutes in total. All eye-tracking data will be collected on Tobii eye- tracking systems.

A.4.3. If subjects are assigned or randomized to study "arms" or groups, describe how they are assigned.

- *Describe the methods of computing the randomization schedule (if any) and maintaining blinding (if any).*
- *Who will perform these computations?*
- *How will you verify each subject's eligibility prior to randomization?*

N/A

A.4.4. Describe any follow up procedures.

There are no current plans for long-term follow-up.

A.4.5. Once this study has been approved by the IRB, for how many months or years will this study be active (you are collecting data or have access to identifiers)?

This study will be active for 4 years.

A.4.6. Will this study use any of the following methods?

- ✔ Audio Recording
- ✔ Video Recording
- ✘ Behavioral observation - (e.g., Participant, naturalistic, experimental, and other observational methods typically used in social science research)
- ✔ Pencil and paper questionnaires or surveys
- ✔ Electronic questionnaires or surveys
- ✔ Telephone questionnaires or surveys
- ✔ Interview questionnaires or surveys
- ✘ Other questionnaires or surveys
- ✘ Focus groups
- ✘ Diaries or journals
- ✘ Photovoice
- ✘ Still photography
- ✘ Unencrypted Messaging with Participants (e.g., text messages, unencrypted emails)

A.4.7. If there are procedures or methods that require specialized training, describe who (role/qualifications) will be involved and how they will be trained.

Intervention: Intervention staff will be project staff from RTI and the UNC TEACCH Autism Program with expertise in early intervention. All will receive training on both phases and regular supervision on implementation of this intervention and from the broader investigative team on Fragile X. For Phase 1 of PIXI, interventionists will receive training on rare neurogenetic disorders, routines-based intervention home visiting models, and sensitive responding from the lead clinician and educational consultants. For Phase 2, interventionists will receive training on implementation of Infant Start intervention. Interventionists who previously implemented iBasis VIPP were trained in the intervention protocol.

Assessments: Assessments will be conducted by licensed psychologists and trained research team members from RTI International with expertise in early child development and neurodevelopmental disorders. Assessors have attended prior training on standardized assessments and will spend 10-20 hours ensuring all assessment protocols are administered with validity and in a standardized method across assessors.

A.4.8. Are there cultural issues, concerns or implications for the methods to be used with this study population?

No

A.4.A. Biomedical methods and procedures

A.4.A.1. Is this an interventional study?

Yes

Distinguish what is being done specifically for this research from procedures that would be done anyway for clinical care:

Delays in diagnosis can reduce timely access to interventions that could have significant consequences for the developmental trajectories of these children. Through the Early Check Screening (IRB #18-0009), children who may typically not have been identified with these disorders until they display symptoms will be able to participate in early intervention services prior to the emergence of symptoms.

Next, assessment and coding procedures that would not be used in standard clinical practice will be used; measuring three broad outcome areas: social validity of PIXI, child development, and parent responsivity and efficacy.

Lastly, weekly family support sessions will be provided immediately following the diagnosis. All of the identified intervention models with any empirical basis have begun the direct intervention with infants after the age of 6 months—most start as late as 9 or 12 months. The Infant Start intervention model has been used with infants as young as 7 months; therefore, to maintain the integrity of that intervention, phase 2 of the intervention will start no earlier than when the infant is 6 months of age. However, per the Early Check protocol (IRB #18-0009), all of the infants will have been screened before 2 months of age, providing a gap of up to 4 months where infants and families will not be receiving formal intervention. It is proposed an initial phase of intervention that will involve regular but less formal contact with the family in the months between diagnosis and the start of the phase 2 intervention.

A.4.A.2. If the study involves the use of placebo control, provide justification

N/A

A.4.A.3. Will this study involve drugs, biologics or other substances (such as a botanical or dietary supplement)? For guidance on dietary supplements, see Section VI, C FDA guidance document UCM229175.pdf

No

A.4.A.4. Is there an Investigational New Drug application (IND) for this study?

No

Please check below:

✔ This study does not involve drugs, biologics or other substances.

✘ I am using a U.S. commercially available agent, consistent with labeling.

✗ I am studying a botanical substance or dietary supplement intended to affect the structure and/or function of the body; it is **not** intended to cure, treat, mitigate, prevent or diagnose disease, including its associated symptoms.

A.4.A.5. When the intent of a clinical investigation is to collect information about the safety or effectiveness of a device, the need for an Investigational Device Exemption (IDE) must be evaluated. Please review the Investigational Device Guidance document prior to completing this section. Your response to the following questions will determine if an IDE is needed.

A. Select the response that best describes your investigation:

✗ 1. This research is **investigator-initiated** and is designed to study one of the following:

- An unapproved device (includes assays [e.g. in vitro diagnostics], software, algorithms and some mobile applications.)
- An approved device with unapproved components
- A new indication for an approved device **even if no marketing application is planned**

✗ 2. This research is designed to support an IDE (device marketing application).

✗ 3. This research is designed to collect safety and/or effectiveness information about a device.

✗ 4. The device(s) in this research is being **used as "tool"** to address a research question, collect information or test a physiologic principle. No data is collected about the device itself.

✓ 5. None of the above.

A.4.A.6. Does your study involve any of the following? (check all that apply)

✗ Embryonic stem cells

✗ Fetal tissue

✗ Genetic testing (see GINA and GWAS)

✗ Clinical laboratory tests

If McLendon Labs will do the testing, you must complete the appropriate form found at UNC Health Care and submit to them for review.

✗ Testing for communicable diseases that have mandated reporting requirements (link to state guidance)

✗ Point of Care Testing (POCT), which is CLIA-approved testing done at the "bedside" or site of care by hospital or clinic personnel (not by subject). Examples include urine pregnancy testing, glucose monitoring, etc.

If McLendon Labs will do the testing, you must complete the POCT form found at UNC Health Care and submit to them for review.

✗ If your study utilizes **radiopharmaceuticals** to address basic science questions, an IND is not necessary.
Instead, your study will be reviewed/approved by the Radioactive Drug Research Committee (RDRC); approval by the Radiation Safety Subcommittee (RSS) is not required.
If you have questions about the RDRC approval process, please contact Dede Corvinus.

✗ Diagnostic or therapeutic ionizing radiation, or radioactive isotopes (not covered under 21 CFR 361.1), which subjects would not receive otherwise if not participating in this research study. Do not check if all radiation is administered as standard of care. Do check if your study includes views/scans that represent no greater than minimal risk as determined by the Radiation Safety Sub-committee (Guidance).
Application for Human Use of Radiation in Research.

Select which option applies to your study:	--

✗ Gadolinium administered as a contrast agent

✗ IBC (Institutional Biosafety Committee) - Recombinant DNA or gene transfer to human subjects

✗ Any research activities conducted in the UNCHC Perioperative areas. This includes Pre-care, Pre-op, Operating room and PACU.
You must complete the Checklist for Perioperative Services and return it to moe_lim@med.unc.edu

✗ Any form of medical imaging (ultrasound, MRI, CT, X-ray, PET-CT, PET-MRI)

A.4.A.7. Will your study involve storage of specimens for future unspecified research?

No

A.5. Benefits to subjects and/or society

A.5.1. Describe how this study will contribute to generalizable knowledge that will benefit society.

Identification of infants with rare neurogenetic disorders at birth through Early Check (IRB #18-0009) affords us an unprecedented opportunity to intervene in ways that could improve developmental trajectories and quality of life for the children and their families. If the benefit of early, pre-symptomatic intervention could be demonstrated for these disorders, the findings would likely have significant generalizability to other conditions that result in intellectual and developmental disabilities, providing critical information to community-based early intervention programs about pre-symptomatic treatment in established conditions, and could expand perspectives regarding the benefit of a including a broader range of conditions on the Recommended Uniform Screening Panel.

A.5.2. Does this study have the potential for direct benefit to individual subjects in this study?

Yes

Consider the nature, magnitude, and likelihood of any direct benefit to subjects. If there is no direct benefit to the individual subject, say so here and in the consent form, if there is a consent form. Do not cite monetary payment or other compensation as a benefit.

Explain

Infants involved in the study will receive intervention that addresses the core deficits seen in children with rare neurogenetic disorders (communication, social, sensory, cognition, motor skills) which may benefit them in the future progression of the condition. Additionally, children will gain access to early intervention pre-symptomatically or as very early symptoms emerge, which may be associated with improved developmental outcomes. The intervention will include parent education about the disorder as well as direct parent coaching around parent-child interactions and repeated comprehensive assessments of family and child functioning. This parent-mediated approach aims to achieve two goals (1) general enrichment of the core interactive social experience for infants and (2) specific attention within this to addressing any emerging atypical behaviors that might be expected at this age and their interactional consequence.

A.5.3. Are there plans to communicate the results of the research OR results of any clinical tests administered for the research back to the subjects?

Yes

If yes, describe

Parents will receive a brief summary report after each of the 6-36-month visits. This will include a summation of standard assessments typically used in clinical care, but presented to parents as findings form a research study and very clearly indicated as non-diagnostic.

Summary results of the larger study will be shared with families through a mailed letter after study completion.

A.6. Risks and measures to minimize risks

For each of the following categories of risk you will be asked to describe any items checked and what will be done to minimize the risks.

A.6.1. Psychological

✓ Emotional distress

✗ Embarrassment

✓ Consequences of breach of confidentiality (Check and describe only once on this page)

✗ Other

A.6.2. Describe any potential psychological risks checked above and what will be done to minimize these risks

There are minimal anticipated risks to participating infants or families.

To minimize the risk of an infant becoming stressed, all assessment and intervention sessions will be conducted in a setting comfortable to the child. Intervention sessions will be conducted in the home. Assessments will be conducted in-home and in a laboratory setting that will be designed to be infant friendly, with appropriate furniture and toys. Parents of the infants will present during the assessments and infants will have access to them if they become distressed.

If an infant presents with an unusually fearful reaction and cannot be easily soothed or redirected, as decided by the interventionist/assessor or the parent, during the developmental assessments or the home intervention sessions the task will be discontinued and resumed only when the infant's behavior indicates an ability to participate without significant fear or frustration. If the infant seems unable to participate due to fear or frustration or the interventionist/assessor or the parent feels the task will be too stressful for the child, the assessment/intervention will be discontinued.

Only personnel who have completed the required human ethics trainings will have access to individuals private, identifiable information. All participants will receive a unique identification number (ID) that will be used for any documents, videos, and materials related to the child. Names associated with the ID will be kept in password-protected document on a secure computer server. All family/child paper and/or electronic data will be stored in a locked cabinet or in an encrypted computer. All video recorders will be stored in a locked cabinet. Videos will be removed and deleted from cameras and stored on a secure server in a timely manner. Materials in transit (i.e. videos, assessment protocols) will be kept in a locked box until securely stored on site. Any scientific reports, article submissions, or summaries of data will not include children/family names. Data will be presented in a summary format that will prevent any personal identification.

Because the LENA (used during PANDABox assessment) records not only the child's vocalizations, but also conversations or other background noise in the child's environment, it is possible that sensitive information may be recorded while the child is wearing the LENA device. Families will be given a data permission form that allows them to erase any section of the day or the whole recording before any additional coding is conducted. Participants are also clearly made aware of this risk in the consent form.

There is a slight risk that the heart rate monitor (used during the PANDABox assessment) will be uncomfortable to the participants. Research assistants will be well trained to help virtually guide parents in needed adjustments to improve comfort. Caregivers will also be assured that they can terminate any portion of the study, including collection of heart rate, at any time.

Reflecting on their infant's functioning during form completion may be distressing for parents. Although they may feel some distress with these activities, we do not anticipate it being any more distressing than what might be expected within the clinical setting.

Plans to ensure necessary intervention in emotional distress. Research team members will follow a protocol for emergency situations and the project will adhere to the state legal statutes governing mandated reporting of suspected child abuse. If, during any contact with a project staff member, a parent expresses suicidal ideation, or is so immobilized by symptoms that she or her child are in danger, or is in danger because of domestic violence, the research team member will assess the mother and contact Drs. Okoniewski, Hazlett, or Wheeler (all licensed psychologists in NC). If the research team member is with the parent, they will remain with them until a safe resolution has been reached, (i.e., immediate referral to a community resource and/or involvement of supporters who can continue to ascertain the safety of the mother and infant/toddler after the team member leaves the premise).

Some infants may show early signs of developmental delay that warrant intervention. If an infant shows signs of developmental challenges, researchers will provide parents with information to follow up with their child's pediatrician for a referral to Part C Early Intervention programming.

There is a slim chance of a breach of confidentiality. RTI and UNC consider the privacy of research

participants to be of the upmost importance.

A.6.3. Social

- ✘ Loss of reputation or standing within the community
- ✘ Harms to a larger group or community beyond the subjects of the study (e.g., stigmatization)
- ✘ Consequences of breach of confidentiality (Check and describe only once on this page)
- ✘ Other

A.6.4. Describe any potential social risks checked above and what will be done to minimize these risks

No Answer Provided

A.6.5. Economic

- ✔ Loss of income
- ✘ Loss of employment or insurability
- ✘ Loss of professional standing or reputation
- ✘ Loss of standing within the community
- ✘ Consequences of breach of confidentiality (Check and describe only once on this page)
- ✘ Other

A.6.6. Describe any potential economic risks checked above and what will be done to minimize these risks.

There may be a possible loss of income due to participation in this project. While the research team will make an effort to accommodate family schedules in intervention sessions and assessments, there may be the rare circumstance where a working parent may need to miss work time not covered by leave or other means. To mitigate potential economic risk, participants will be compensated $50 for each assessment visit.

A.6.7. Legal

- ✔ Disclosure of illegal activity
- ✔ Disclosure of negligence
- ✘ Consequences of breach of confidentiality (Check and describe only once on this page)
- ✘ Other

A.6.8. Describe any potential legal risks checked above and what will be done to minimize these risks

While participants and families will not be asked to disclose any illegal activity or negligence, research staff will spend a significant time interacting with families. Assigned interventionists will interact with families for at least 24 weeks for 1-2 hours per week through video calls and sessions in the home. Due to the length of interaction with families and the provision of services in the home, interventionists and assessors may become aware of such information. Project staff will be aware of their legal obligations to report any suspected cases of child abuse or neglect to the appropriate authorities.

A.6.9. Physical

- ✘ Medication side effects
- ✘ Pain
- ✔ Discomfort
- ✘ Injury
- ✘ To a nursing child or a fetus (either through mother or father)

A.6.10. Describe any potential physical risks checked above, including the category of likelihood and severity, and what will be done to minimize these risks. Where possible, describe the likelihood of the risks occurring, using the following terms:

- Very Common (approximate incidence > 50%)
- Common (approximate incidence > 25 - 50%)
- Likely (approximate incidence of > 10 - 25%)
- Infrequent (approximate incidence of > 1 - 10%)
- Rare (approximate incidence < 1%)

Describe severity of risks using the following grading scale:

- Mild- No disruption to the subject's ability to perform daily activities; may include non-prescription intervention only
- Moderate- Temporary interference with daily activities; may include prescription intervention
- Severe- Interference with daily activities; medically significant but not life threatening
- Life threatening

Examples:

Rare (< 1%) and Severe: blindness
Rare (< 1%) and Mild: dry skin, dry mouth, transient headache

If you are using these terms differently than described above, please provide your study-specific definitions.

Phase 1 trials: Due to limited experience, incidence may be better described as the number of events that have occurred in the total number of animals/humans studied.

Participants might feel discomfort while wearing the LENA vest and/or heart rate monitors. All efforts will be made to reduce discomfort; however, if participants don't reach a point of comfort during the assessment hey will be allowed to remove the LENA vest and/or heart rate monitor at any time.

A.6.11. Unless already addressed above, describe procedures for referring subjects who are found, during the course of this study, to be in need of medical follow-up or psychological counseling

During Phase 1 of PIXI, the therapist will provide general education about the baby's disorder, support, and referrals for services as warranted by the family's expressed concerns.

A.6.12. Are there plans to withdraw or follow subjects (or partners of subjects) who become pregnant while enrolled in this study?

No

A.7. Data and safety monitoring

A.7.1. When appropriate, describe the plan for monitoring the data to ensure the safety of participants. These plans could range from the investigator monitoring subject data for any safety concerns to a sponsor-based data and safety monitoring board or committee (DSMB, DSMC, DMC), depending on the study. For studies that do not raise obvious safety concerns, you may still describe your plans for monitoring the study as it progresses.

Although this study presents minimal anticipated risk, the study PI will be responsible for the safety of all participants and the validity and integrity of the data. The PIs will regularly monitor the study and data collection procedures and conduct team meetings to review the protocols for safety and confidentiality.

A.7.2. If not already addressed above, describe the plans for aggregate review of unanticipated problems (including but not limited to adverse events) across all sites, in order to monitor subject safety.

No Answer Provided

A.7.3. What are the criteria that will be used to withdraw an INDIVIDUAL SUBJECT from this study or halt the research intervention (e.g., abnormal lab tests, allergic reactions, failure or inability to comply with study procedures, etc.)?

While this study has no plans to withdraw individual subjects, if subjects are unable to attend intervention sessions or frequently cancel appointments participants may not be able to fully complete the intervention within the allotted time frame. If cancellations are frequent, a member of the study team will inform the participant of an intervention end date in order to accommodate further enrollment of participants. Subjects may voluntarily withdraw.

A.7.4. Are there criteria that will be used to stop the ENTIRE STUDY prematurely (e.g., safety, efficacy, unexpected adverse events, inability to recruit sufficient number of subjects, etc.)?

No

A.7.5. Will this study involve a data and safety monitoring board or committee?

No

A.8. Data analysis

A.8.1. Summarize the statistical analysis strategy for each specific aim.

For this case series, analyses will be primarily descriptive. Researchers will first examine feasibility and acceptability and descriptive data on pre-post measures at the case level. Next, change scores on each measure over the intervention period will be reviewed to help identify primary outcome measures and effect sizes for a larger trial. Finally, the team will compare developmental and behavior profiles of this sample at age 3 to those of young children with the disorder who were not identified through Early Check (IRB #18-0009).

A.8.2. If this is a pilot study, please describe the future study and say how its study design, aims, sample size, and methods differ from the pilot study you are proposing.

Upon completion of the case series, the team will use data collected on family needs, acceptability and feasibility of the intervention, child skills, parent responsivity, and social validity to refine (1) PIXI and develop a formalize treatment manual for all components, (2) our hypotheses about treatment effects, and (3) our measurement plan. A meeting will be held with consultants and Early Check expert advisors during year 2 of this project to review results and plan next steps. It is anticipated revisions to include manualizing the content and strategies incorporated into parent calls, timing and telehealth revisions, possible adaptations to phase 2 necessary for unique needs of those with rare neurogenetic disorders, and development of fidelity measures for all components.

Although final decisions regarding next steps will be decided after completion of the case series, it is expected that the next stage of intervention development will be to implement the revised PIXI intervention in a preliminary efficacy wait-list control design trial. In this trial, the plan is for all families to begin phase 1 after diagnosis. Based on feedback from families, the addition of formal environmental enrichment components such as increased motor and language stimulation during phase 1 will be considered. Families will be randomly assigned to early phase 2 or delayed phase 2 (e.g., 3-month delay). After completion of all the components of PIXI, all families will return for posttest. The team anticipates measuring the same three areas as in the case series: child outcomes, parent responsivity, and social validity. Outcome measures will be completed and coded by a team who is blind to group assignment and to ongoing therapeutic conversations to reduce bias. Treatment fidelity of the manualized PIXI will also be measured.

A.8.3. Provide a compelling justification for the proposed sample size in terms of the likelihood of achieving each aim.

One of the challenges in designing an effective intervention for infants with rare neurogenetic disorders identified through Early Check (IRB #18-0009) is determining an appropriate comparison group for assessing efficacy of our intervention. Even with the most liberal of estimates of uptake for the Early Check program, we would still only anticipate identifying around 20 infants per year. Therefore, even in the best-case scenario, we would not have enough participants to power a true randomized control trial (RCT) of the intervention. To address this issue for the FX sample, we are proposing several activities, all of which are already underway.

First, we are currently working on a collaborative, multisite paper pooling early developmental data collected on young children with FXS. This paper will be based on the Mullen Scales of Early Development (Mullen), administered to over 300 children with FXS under the age of 5 (with an estimated 900+ assessment points) to provide an overview of the extent of delay in five core developmental areas (Cognition, Fine Motor, Gross Motor, Receptive Language, Expressive Language) for boys and girls with FXS under the age of 5. This data will provide a general estimate of functioning at discrete ages to serve as a comparison for those who are identified at birth with FXS and complete the PiXI.

Second, we will publish findings from a soon-to-be-launched survey on parent reports of early development and access to and dosages of early intervention services for a national sample of children under the age of 7 with FX. This will provide additional information about early experiences of young children with FX by which to compare the experiences of our Early Check sample.

And lastly, we will identify, through the RTI Our Fragile X World registry, as well as by working with North Carolina-specific FX advocacy groups (NC CSN and consultant Kerry McCarthy Adams), a sample of children under 5 with FX who have lived in North Carolina for most of their lives (n = ~15). We will recruit these children and their families into a study about early development of children with FX in North Carolina and will collect direct assessments of these children as well as parent perspectives on their child's access to and dosage of early intervention services in the state. Although this comparison cohort will still be limited by a historical confound, this sample will be our closest possible group to our Early Check (IRB #18-0009) sample.

A.8.4. Summarize the plans for data management.

Assessment data will be entered and managed via REDcap by RTI research team members. Physical assessment protocols will be deidentified and securely stored at RTI. Video and audio files will be stored on a secure server at UNC or RTI.

The security standards for the protection and integrity of all data are described in Section A.10 and include storage on a HIPAA-compliant server in the RTI Enhanced Security Network, two-factor authentication, and back-up of the data performed by RTI ITS daily. Data access will be limited only to those team members who require access to perform data entry and analysis.

Regular review and data checks will be performed throughout all data collection and analysis activities in order to prevent, identify and correct any errors. All data analysis activities will be performed independently by two team members and compared for accuracy. The Principal Investigator is responsible for the final review of all analyses.

A.9. Identifiers

A.9.1. Check which of the following identifiers you already have or will be receiving, or select "None of the above."

- ✔ Names (this would include names/signatures on consent forms)
- ✔ Telephone numbers
- ✔ Any elements of dates (other than year) for dates directly related to an individual, including birth date, admission date, discharge date, date of death. For ages over 89: all elements of dates (including year) indicative of such age, except that such ages and elements may be aggregated into a single category of age 90 and older
- ✔ Any geographic subdivisions smaller than a State, including street address, city, county, precinct, zip code and their equivalent geocodes (e.g. GPS coordinates), except for the initial three digits of a zip code
- ✘ Fax numbers
- ✔ Electronic mail addresses
- ✘ Social Security numbers
- ✘ Medical record numbers
- ✘ Health plan beneficiary numbers
- ✘ Account numbers
- ✘ Certificate/license numbers
- ✘ Vehicle identifiers and serial numbers (VIN), including license plate numbers
- ✘ Device identifiers and serial numbers (e.g., implanted medical device)
- ✘ Web universal resource locators (URLs)
- ✘ Internet protocol (IP) address numbers
- ✘ Biometric identifiers, including finger and voice prints
- ✔ Full face photographic images and any comparable images

❌ Any other unique identifying number, code, or characteristic, other than dummy identifiers that are not derived from actual identifiers and for which the re-identification key is maintained by the health care provider and not disclosed to the researcher

❌ None of the above

A.9.2. For any identifiers checked, how will these identifiers be stored in relationship to the research data?

❌ with the research data (i.e., in the same data set and/or physical location)

✔ separate from the research data (i.e., coded with a linkage file stored in a different physical location)

Provide details about the option you selected above:
Identifiers will be stored separately from research data in a password protected document linking identifying information to a participant's ID number.

A.9.3. Are you collecting Social Security Numbers to be used as a unique identifier for study tracking purposes for national registry or database? (Do not check yes if collecting SSN *only* for payment purposes; this will be addressed later.)

No

A.10. Confidentiality of the data

A.10.1. Describe procedures for maintaining confidentiality of the data you will collect or will receive (e.g., coding, anonymous responses, use of pseudonyms, etc.).

Only personnel who have completed the required human ethics trainings will have access to individuals private, identifiable information. All participants will receive a unique identification number (ID) that will be used for any documents, videos, and materials related to the child. Names associated with the ID will be kept in password-protected document on a secure computer server. All family/child paper and/or electronic data will be stored in a locked cabinet or in an encrypted computer and identified only by child ID. All video recorders will be stored in a locked cabinet. Videos will be removed and deleted from cameras and stored on a secure serve in a timely manner. Any scientific reports, article submissions, or summaries of data will not include children/family names. Data will be presented in a summary format that will prevent any personal identification.

A.10.2. Describe how data will be transmitted among research team (i.e., personnel listed on this application).

Questionnaire data will include only ID numbers and be entered directly into secure REDCap database systems. Any hard copy data will be transported directly to a UNC or RTI office in a confidential portable file in a locked file box and entered in the office for access by the team. De-identified data only will be transmitted within the research team by password protected, encrypted email. Access to the data files will be restricted, based on the role of the research team member.

A.10.3. Are you collecting sensitive information such as sexual behavior, HIV status, recreational drug use, illegal behaviors, child/physical abuse, immigration status, etc?

No

A.10.4. Do you plan to obtain a federal Certificate of Confidentiality for this study? Please note that all ongoing or new research funded by NIH as of December 13, 2016 that is collecting or using identifiable information is automatically issued a Certificate of Confidentiality (CoC). You should also select “Yes” if your study is NIH funded and has been issued a CoC under this updated NIH policy.

NOTE: Investigators utilizing ANY federal funding to conduct this research should review the COC website to determine if their funding agency issues COCs automatically (as the NIH does) or if they might need to apply for the COC via the online COC system. Unfunded and non-federally funded investigators may also apply for a COC via the online COC system.

No

A.10.5. If this study is limited to data collection by survey or interview, discuss the potential for deductive disclosure (i.e., directly identifying subjects from a combination of indirect IDs).

The primary potential for deductive disclosure will be through videotaping of research participants. Videotapes

of intervention sessions will be viewed and coded for parent responsivity. Videotapes will have full face images of children and family and it is likely first names will be used throughout the interaction with interventionist, creating a potential for identification. We, therefore, will take careful measures to protect the security of videotapes. The IDs used for other assessments and materials offer little potential for deductive disclosure.

A.10.6. Will any of the groupings or subgroupings used in analysis be small enough to allow individuals to be identified?

Yes

Describe these groupings and sample sizes projected.

There is a possibility that participating individuals could be identified based on knowledge of infants who have a confirmed positive diagnosis of a rare disorder. Even with the most liberal of estimates of uptake for the Early Check program (IRB #18-0009), we would still only anticipate identifying around 10-20 infants per year.

A.11. Data sharing and transmission

A.11.1. Check all of the following who will receive **identifiable data** (contains any of the 18 identifiers listed above) outside the immediate research team (i.e., not listed as personnel on this application)? *

- ✗ No one
- ✗ Coordinating Center
- ✗ Statisticians
- ✗ Consultants
- ✗ Other researchers
- ✗ Registries
- ✗ Sponsor and/or its designee(s)
- ✗ External labs for additional testing
- ✗ Journals
- ✗ Publicly available dataset
- ✓ Other

If other, please specify.

Dr. Vismara, educational consultant, will receive video footage of Phase 2 intervention sessions. Within the video, Dr. Vismara will be able to see the faces of the parent-infant dyad. It is possible that identifiable information (i.e. child name) may be disclosed within the video but the research team will share no identifiable information with Dr. Vismara outside of what is disclosed within the video. Dr. Vismara's contract with RTI International details confidentiality agreements and is attached.

A.11.2. For any recipients checked above, explain the confidentiality measures to be taken

All confidential information made available, disclosed, or otherwise known to Dr. Vismara as a result of her contract with RTI shall be considered the sole property of RTI and/or RTI's study participants. Dr. Vismara may only use confidential information for the purposes of performing the services or obligations hereunder. Both during the terms of the contract and at all times thereafter, Dr. Vismara shall not reveal, publish or otherwise disclose confidential information to any third party without prior written consent of RTI.

A.12. Post-study disposition of identifiable data or human biological materials

A.12.1. Describe your plans for disposition of data or human biological specimens that are identifiable in any way (directly or via indirect codes) once the study has ended. If you plan to destroy linkage codes or identifiers, describe how and when this will be done.

Hard data will be shredded and sent to a qualified handler of confidential documents and digital data will be removed from the secure server at the end of the study.

Part B. Direct Interaction

B.1. Methods of recruiting

B.1.1. Check all the following means/methods of subject recruitment to be used:*

- ✔ In person
- ✘ MyChart

To utilize MyChart for research recruitment purposes, please complete the form (click here), and upload a PDF copy of the completed MyChart request to your application.

- ✘ Participant pools
- ✘ Presentation to classes or other groups
- ✘ Letters
- ✔ Flyers
- ✘ Radio, TV recruitment ads
- ✘ Newspaper recruitment ads
- ✘ Website recruitment ads
- ✔ Telephone script
- ✔ Email or listserv announcements
- ✘ Follow up to initial contact (e.g., email, script, letter)
- ✘ N/A
- ✔ Other

If other, please specify

Informational page to be given to families at in person consent meeting. Interested participants will be directed to the webpage contact form to confirm eligibility.

B.1.2. **Research for Me @UNC**
A comprehensive study listing and engagement site intended to fulfill the mission of improving transparency and awareness of research at UNC.
All study involving direct interaction with participants must be listed on this site; you may choose whether to further utilize your listing for participant recruitment.
Instructions:

- Choose Basic or Recruitment
- Click on link to open listing submission form in a new tab
- Submit online form

✔ **Basic Listing (Click here to open basic submission form)**

For studies that do not want to be contacted by potential participants. Submit very basic information in lay language, but no details or team contact information will be displayed.
Exception from this kind of listing is rare, but may be requested for consideration via this form.

✘ **Recruitment Listing (Click here to open recruitment submission form)**

For studies that want to utilize the free recruitment features of the website. Participants can view more details about your study and contact the team to express interest.
You control the timeframe for display. Get a unique URL and QR code for use on other materials. Site is promoted to patients and the public by NC TraCS.

View examples, manage submitted listings, find FAQ, and download PDFs at researcherdashboard.unc.edu
Please direct all questions and feedback to Research for Me

B.1.3. Describe how subjects will be identified

Subjects will be identified through the Early Check Program (IRB #18-0009). Early Check is a voluntary research program in which newborns will be screened for a carefully selected panel of rare neurogenetic conditions. It will be offered under a research protocol with parental permission. Early Check is a complement to the state-mandated newborn screening and is conducted in collaboration with the North Carolina State Laboratory of Public Health, utilizing existing newborn blood spots to test for these conditions. Infants diagnosed through Early Check will be invited to participate in the intervention. Additionally, eligible self-referrals may be invited to enroll in the study if slots are available (see B.1.6).

Self-referrals may be identified using announcements/flyers posted on social networking sites and sent via parent/advocacy groups, group list serves and the National Fragile X Foundation. Recipients will be asked to share the announcement with others who may be eligible and interested.

B.1.4. Select any of the following procedures solely conducted for screening, recruiting, or determining the eligibility of prospective human subjects. (Note: you should only collect the minimal information needed for these purposes.)

✔ Obtain information through oral or written communication with the prospective subject or legally authorized representative
This includes online, telephone, or in-person screening questionnaires or interviews.

✘ Obtain already collected identifiable private information or records
Examples include review of medical charts, data repositories, and administrative records.

✘ Reviewing/testing identifiable biospecimens by accessing stored biospecimens and related information

✘ None of the above

B.1.5. For any selections made, please describe the procedures. (Respond “N/A” if “None of the above” is selected.)

Screenings will occur for families that self-refer to the study. When a family or provider of the family (i.e. pediatrician) contacts the study team in person, by email, telephone, or online they will be asked for a copy of the genetic report confirming the diagnosis, the age of the infant's diagnosis, the current age of the infant, and the state and county where the family resides.

B.1.6. For any information collected for these purposes, please describe when and how you will destroy the data if the participant declines to participate or is not eligible. (Respond “N/A” if “None of the above” is selected.)

If the participant declines to participate or is not eligible any hard copy data will be shredded and online contact forms deleted. Phone calls will not be recorded and therefore the information will not be stored.

B.1.7. Describe how and where subjects will be recruited and address the likelihood that you will have access to the projected number of subjects identified in A.2.

Subjects will be recruited during the Early Check (IRB #18-0009) genetic counseling/confirmatory testing process. We will discuss with parents the intervention and provide them with a simple overview of the study. Parents who choose to defer enrollment will be called one week after their final genetic counseling visit and asked if they would like to participate. Additionally, families may self-refer to the study and contact the research team. If slots are available based on the age of their baby and the current number of infants receiving intervention, eligible self-referrals will receive an informational handout about PiXI (see attached). All families with infants diagnosed through Early Check will be invited to enroll in PIXI. Due to the limited number of interventionists, invitations to families with FXPM infants and self-referred families will be prioritized as slots become available according to the following priority list:

1. Infants with a confirmed diagnosis identified through Early Check (IRB #18-0009)

2. Infants with FXS whose families self-refer to the study

3. Infants with premutation fragile X (PMFX) identified through Early Check (IRB #18-0009) who show signs of delay at their 6-month assessment

4. Infants with PMFX identified through Early Check (IRB #18-0009) who don't show signs of delay at their 6-month assessment but may show increased signs of genetic risk or whose parents show increased anxiety regarding their child's development

If invited families choose to enroll, the research team will schedule a baseline assessment.

Even with the most liberal of estimates of uptake for the Early Check program, we would still only anticipate identifying around 10 eligible infants per year.

B.1.8. Describe how you will protect the privacy of potential subjects during recruitment

In-person recruitment will occur in a private room during the genetic counseling visit to protect the privacy of potential subjects.

Recruitment via the phone will occur in a private office with the door closed to protect the privacy of potential subjects.

The online contact form is housed on the secure EarlyCheck Portal at RTI.

B.1.9. Describe how subjects will be contacted, if not addressed above

Participants will be contacted in-person, by email, and/or by phone.

B.1.10. Describe who (by role) will do the recruiting

Phone contacts and recruitment scheduling will be the responsibility of the research coordinator, research assistants, or lead investigators.

B.1.11. Describe efforts to ensure equal access to participation among women and minorities

We expect eligible participants to reflect the demographics similar to that of the diagnosed population. However, parents must be fluent in English to participate, a requirement that may decrease the number of Hispanic families eligible.

B.2. Protected Health Information (PHI)

Protected Health Information (PHI) is any identifiable information about the subject's health that relates to their participation in this research and is obtained from sources other than the subject, such as medical records, health care providers, insurance plans, etc. more

B.2.1. Are you requesting a limited waiver of HIPAA authorization?
If you need to access Protected Health Information (PHI) to identify potential subjects who will then be contacted, you will need a limited waiver of HIPAA authorization (see SOP 1801, 2.3). This does not apply to situations where you will never contact subjects directly (e.g., retrospective chart review), in which case you should request a full waiver under section D.

No

B.2.2. Will you need ongoing access to PHI (e.g., medical records) to conduct the study, beyond the identification of potential subjects as addressed above? In this case you will need to obtain a signed HIPAA Authorization from each subject.

No

B.3. Subject Contact, Duration and Privacy

B.3.1. Number of contacts per subject (contacts includes in-person, telephone, email, mailings, etc.)

Depending upon the age of entry, the study team will have anywhere from 40 to 60 contacts with participants.

B.3.2. Duration of each contact. If multiple contacts, provide the range or average time for each contact.

Up to 5 years

B.3.3. Total duration of individual subject's participation, including follow up evaluation, if applicable

3 years

B.3.4. Where are you studying subjects or obtaining their data?

- ✔ Non-healthcare setting
- ✔ Healthcare setting

Please check all that apply:

- ✔ UNC Medical Center (N.C. Memorial, N.C. Children's Hospital, N.C. Womens' Hospital, N.C. Cancer Hospital, N.C. Neurosciences Hospital, Hillsborough Campus, Ambulatory Care Center (ACC), UNC School of Medicine, UNC Eastowne)
- ✘ Caldwell Memorial Hospital
- ✘ Chatham Hospital
- ✘ Johnston Health (FWA# 1764)
- ✘ Nash Health Care (FWA# 14328)
- ✘ Onslow Memorial Hospital (FWA# 12614)
- ✘ Pardee Hospital (FWA# 2918)
- ✘ UNC Lenoir Health Care (FWA# 7114)
- ✘ UNC Physicians Network (FWA# 30031)
- ✘ UNC Rex Healthcare (FWA# 740)
- ✘ UNC Rockingham Health Care (Morehead Memorial Hospital FWA# 24488)
- ✘ Wayne Memorial Hospital (FWA# 2782)
- ✘ Other

B.3.5. Describe procedures that will ensure privacy of the subjects in this study. Examples include the setting for interviews, phone conversations, or physical examinations; communication methods or mailed materials (e.g., mailings should not indicate disease status or focus of study on the envelope)

Any phone conversations will be conducted in private offices.

Assessments will be conducted in private treatment rooms or in the family home.

Video conferencing calls between interventionist and caregiver will use a secure video conferencing system. Interventionists will complete calls behind a closed door in a private office. Caregivers may choose where they receive their calls and will be informed that if they are to complete the call publicly they are at risk of others overhearing.

Intervention sessions will occur in the privacy of the family home or at an alternative private location.

B.3.6. Provide more information about the location(s) where research will be conducted (e.g., if UNC Medical Center is checked in #4 above and study visits will be conducted in the CTRC, enter "CTRC" here.)

Research will take place in participant's homes as well as at the UNC School of Medicine Carolina Institute for Developmental Disabilities (CIDD) and UNC School of Medicine TEACCH Autism Program (TEACCH). Phase 1 and 2 of intervention sessions will occur virtually through video conferencing calls and several in-home visits. Assessments will be administered at the CIDD, TEACCH Centers, or in family homes. Additionally, assessments and intervention sessions may occur at an alternative private location that is convenient to both the participant and the researchers.

B.4. Incentives for participation

B.4.1. Are there incentives (monetary or non-monetary) for subjects to participate or are you reimbursing subjects for study-related costs (e.g., travel, parking, hotel accommodations or childcare)?

Yes

A. Please describe any incentives and/or reimbursements for study-related costs separately below.

Families will receive $50 compensation in the form of a gift card for each assessment visit, excluding assessment 1 completed at the Early Check genetic counseling appointment (IRB #18-0009). Payments will be processed through RTI. Families may also receive travel reimbursement for gas mileage and hotel fees up to $200.

B. Specify the schedule for incentives and if/how this will be prorated if the subject withdraws (or is withdrawn) from the study prior to completing it.

Participants will receive incentives after the completion of an assessment in the form of a gift card. Participants will receive travel reimbursements in the mail after the amount of reimbursement is calculated and processed by RTI. If a participant drops out of the study they will receive incentives for any assessments they have completed but not for any future assessments.

C. For compensation in foreign currency, provide a US dollar equivalent.

Compensation is in the form of US dollars.

D. Discuss the potential for coercion, given factors like the amount of the incentive, the age of the subjects, the purchasing power in foreign countries, the time involved and complexity of procedures, etc.

The incentive is intended to cover the parents'/guardians' time for study participation and incidental expenses for each visit. The participants will receive an estimated $250 in incentives for approximately 15 hours of assessment. According to data released in 2018 by the NC Department of Commerce, the average hourly wage in NC is $22.15. Thus the incentive offered for assessment visits is in-line with what an average wage-earner would get for this amount of time. No incentives are planned for the in-home intervention sessions. Participants will be provided with reimbursement for travel

E. If the subjects are children who will receive the compensation, i.e., the child, the parents or both?

Both children and their parents will participate in our study. Incentive payments will made to the parents.

B.4.2. Are you collecting Social Security numbers or ITIN for payment and/or tax-related purposes?

No

B.5. Costs to be borne by subjects

B.5.1. Will there be any costs that subjects will incur related to participation in the study? Do not include costs for standard care for which patients would be billed if they were not in this study. Also do not include the time spent participating in the study.

Yes

If yes, please check all that apply:

- ✗ Child care
- ✓ Travel
- ✗ Parking
- ✗ Clinic fees
- ✗ Diagnostic tests
- ✗ Laboratory tests
- ✗ Drugs
- ✗ Devices
- ✗ Increased operating room or anesthesia time
- ✗ Other

Please explain any items checked above

Although we will reimburse travel and hotel expenses for families, it is possible that additional travel related costs will incur.

Part C. Existing Data, Records, Specimens

C.1. Data Sources

C.1.1. What existing records, data or human biological specimens will you be using? (Indicate all that apply or select 'None of the above'):

✘ Medical records in any format.

ALERT: You must check both boxes: 1) Medical records in any format and 2) Electronic medical record using Epic, or you/your study team will not be granted access to Epic for research purposes.

✘ Electronic medical records using Epic, WebCIS or other electronic system

✘ Carolina Data Warehouse for Health (CDW-H) (for UNC and its affiliates only)

✘ Carolinas Collaborative Data Request and Review Committee (DRRC)

✘ Paper medical records

If you access the medical records of fewer than 50 patients under a full or limited waiver of HIPAA, submit a copy of your IRB approval letter and a completed Research Disclosure Form to Health Information Management (HIM). Do not submit this information to the IRB. For additional information about this process, you should contact HIM directly at : 919-595-5591 or 919-966-1225 or 919-595-5580.

✔ Data already collected from another research study

Were the investigators for the current application involved in the original collection?	Yes

✘ Patient specimens (tissues, blood, serum, surgical discards, etc.)

Has the clinical purpose for which they were collected been met before removal of any excess?	--

✘ Data already collected for administrative purposes

✘ Student records (You will need to satisfy FERPA requirements: see SOP 3101, section 3.1 for guidance)

✘ UNC Dental Records

✔ Data coming directly from a health plan, health care clearinghouse, or health care provider?

✘ Publicly available data

✔ Other

✘ None of the above

For EACH data source checked above, provide a description of the data, proposed use, how data were collected (including consent procedures), and where data currently reside.

We will use data collected for the Early Check Study (IRB #18-0009). The data collected will include assessments, demographic data, and their diagnostic status. Data will be used to confirm eligibility for the study and to provide a baseline before the onset of intervention protocol. Data was collected by the Early Check team. The data currently resides on site at RTI. We will collect genetic reports to confirm diagnosis and developmental assessments, if applicable, for non-Early Check participants.

C.1.2. Describe your plans for obtaining permission from the custodians of the data, records or specimens (e.g., pathology dept, tissue bank, original researcher):

As a part of the consent process for Early Check (IRB #18-0009), participants will consent to allowing researchers to share assessment and demographic information between related research studies. Research team members of the current study are also IRB approved members of the Early Check study.

For eligible self-referrals, we will obtain relevant assessment data from outside providers. See attached HIPAA form and authorization to disclose information form, "Generic_EI_Authorization_to_Disclose".

C.1.3. Do the custodians of the data, records or specimens require a data use agreement?

No

C.2. Coding and Data Use Agreements

C.2.1. When you receive these data, records or human biological specimens will they be coded? Coded means identifying information that would enable the research team to readily ascertain the individual's identity has been replaced with a number, letter, symbol, or combination thereof (i.e., a code). If you will not be using existing materials, check "No."

No

Part D. The Consent Process

D.1. Obtaining informed consent from subjects

The standard consent process is for all subjects to sign a document containing all the elements of informed consent, as specified in the federal regulations. Some or all of the elements of consent, including signatures, may be altered or waived under certain circumstances. If you will be requesting a waiver answer "not applicable" for any of the following questions that will not pertain to this study. You will be asked to provide relevant information in the section below on waivers.

D.1.1. Will children under the age of majority in their locale (18 years in NC) be enrolled? (Note: Any minor subject who attains the age of majority during the course of the research study must provide consent as an adult, unless consent has been waived, which is requested in section D.3.1.)

Yes

Explain the process for obtaining consent from the subject, parental permission and/or minor assent as applicable (unless a waiver of permission will be requested later) in the sections below. The informed consent process should include the following:

- Provide the participant/parent/LAR with:
  - Information about the study in a language they understand
  - An opportunity to ask questions and have their questions answered
  - Adequate time to consider study participation
  - A signed copy of the consent form (a copy is acceptable)
- Avoid exculpatory language and undue influence.
- Document the consent process in the research record; if consent takes place on the same day as study procedures, document that informed consent was obtained prior to initiating any research-related procedures.

When explaining the process for obtaining consent/assent below, please incorporate the above information. (e.g., do not simply state that the participant will sign the form). The assent process should be developmentally appropriate and provide opportunities for children to discuss their willingness or unwillingness to participate. If assent is required, a child's dissent (unwillingness to participate) MUST be honored.

Study information and consent forms will be presented to families during the genetic counseling appointment. For families that self-refer to the study, consent forms will be presented at the baseline assessment appointment. A research team member will review the consent form with parents and allow time for them to ask questions. If parents should decide not to consent at the genetic counseling appointment, they will be provided with contact information for the research team if they decide to participate at a later point. Families that wish to further contemplate enrollment in PIXI after their genetic counseling appointment will be called a week after their appointment to assess any further interest in the study. Those families who decide to participate in the study after the genetic counseling appointment will review the consent form with an interventionist prior to beginning their first intervention session.

Eligible self-referrals may qualify for over-phone review of the consent form. If such an instance occurs, the participant will be emailed or mailed a copy of the consent form. A member of the research team will review the consent form over the phone or via a telehealth call with the potential participant, allowing ample time for questions and review. If the potential participant agrees to enroll in the study, they will sign the consent form and mail it back to the study team in a provided envelope. Upon receiving the signed consent, the research member who obtained consent will sign the form dating both the day consent was obtained and the date the physical consent form was received.

Check the characteristics of children to be enrolled: *

- ✔ 0 - 6 years
- ✘ 7 - 14 years
- ✘ 15 - 17 years

Explain the process for obtaining the assent of the child (unless waiver of assent will be requested, in which case you should provide justification here).

Children are under the age of 9 months when the initial consent for participation is obtained from their parents.

D.1.2. Will adult subjects be enrolled in your study?

Yes

Explain the process for obtaining consent from the subject.

We will obtain written consent from parents/caregivers for their child's participation in assessments and intervention sessions.

D.1.3. Will decisionally-impaired subjects be enrolled in your study? (includes unconscious patients, some psychiatric disorders, others who lack the capacity to give consent)

No

D.1.4. Are you planning to obtain consent from any Non-English speaking subjects?

No

D.1.5. Describe who (by role) will be obtaining consent or parental permission.

Both team members from the Early Check (IRB #18-0009) and PIXI team will be obtaining consent from participants.

D.1.6. Discuss the potential for influencing the subject's decision to participate. Describe steps that will be taken to minimize undue influence during the consent process. These might include a waiting period between the initial consent discussion and obtaining consent, or obtaining consent by someone other than a person with perceived authority (e.g., professor, employer, treating physician).

Families will be provided information regarding the study prior to obtaining consent, during recruitment. Most infants recruited for the study will have received a diagnosis through the Early Check Program (IRB #18-0009). Eligible families may self-refer to the study. Families will have ample time to review the consent form with a research team member and will be given the opportunity to later decide to participate in the study. Regardless of their decision to participate, families will be provided with resources for support in their community.

D.1.7. Has the sponsor of this study provided a model consent form?

No

D.2. Waiver of written documentation of informed consent

The default is for subjects to sign a written document that contains all the elements of informed consent. Under limited circumstances, the requirement for a signed consent form may be waived by the IRB. For example, this might occur for phone or internet surveys, when a signed consent form is either impractical or unnecessary, or in circumstances where a signed consent form creates a risk for the subject.

D.2.1. Are you requesting a waiver of any aspect of written (signed) documentation?

No

D.3. Full or partial waiver of consent

The default is for subjects to give informed consent. A waiver might be requested for research involving only existing data or human biological specimens. More rarely, it might be requested when the research design requires withholding some study details at the outset (e.g., behavioral research involving deception). In limited circumstances, parental permission may be waived. This section should also be completed for a waiver of HIPAA authorization if research involves Protected Health Information (PHI) subject to HIPAA regulation, such as patient records.

D.3.1. Are you requesting any of the following:

- ✗ a waiver of informed consent in its entirety
- ✗ a waiver or alteration of some of the elements of informed consent
- ✗ a waiver of HIPAA authorization (If you are accessing patient records for this research, you must also request a waiver of HIPAA authorization)

D.3.2. If your request for a waiver applies to some but not all of your subject groups and/or consent forms, please describe and justify

No Answer Provided

D.3.3. Does this request for waiver support a study design that involves deception or withholding of information?

No Answer Provided

Consent Forms

This submission requires the following consent forms

Template Type
Adult Consent Form<br>**I am not using this template because:** Not Yet Available / Not Applicable
Parental Permission Form

This submission includes the following consent forms

File Name	Document Type
HIPAA_Authorization_1.docx	HIPAA Authorization
HIPAA_Authorization_genetic_report.docx	HIPAA Authorization
Adult_Consent_and__Parent_Permission_Form_03312021.docx	Parental Permission Form
LENA Data Use Permission Form.docx	Parental Permission Form

view consent forms

Attachments

This submission requires the following attachments

Document Type
Grant Application
Lead Site/Coordinating Center addendum
Pencil and Paper Questionnaire Survey
Electronic Questionnaire Survey
Telephone Questionnaire Survey
Interview Questionnaire Survey
Flyer for Recruitment
Telephone Script for Recruitment
Email or Listserv Recruitment<br>**This attachment not provided because:** Not Yet Available / Not Applicable
Other Materials for Recruitment

This submission includes the following attachments

File Name	Document Type
Grant_application.pdf	Grant Application
PIXI_recruitment_announcement_03232021.docx	Flyer for Recruitment
PiXI_informational_handout_032321.docx	Other Materials for Recruitment
Telephone_script_EC_delayed_enrollment_03232021.docx	Telephone Script for Recruitment
Telephone_script_full_mutation_self-referral_recruitment_03232021.docx	Telephone Script for Recruitment
PIXI webpage and eligibility form on Eary Check Portal.docx	Website for Recruitment
BRIEF-A Self_1.pdf	Electronic Questionnaire Survey
Edinburgh.pdf	Electronic Questionnaire Survey
IBQ-R_Very Short_1.doc	Electronic Questionnaire Survey

M-CHAT_1.pdf	Electronic Questionnaire Survey
MOS-SSS.docx	Electronic Questionnaire Survey
NeoEAT_Bottle_2018-11-9_1.pdf	Electronic Questionnaire Survey
NeoEAT_Breast_2018-11-9_1.pdf	Electronic Questionnaire Survey
PHQ-9.pdf	Electronic Questionnaire Survey
PSI_Placeholder.pdf	Electronic Questionnaire Survey
Pedi-EAT_Full_Version_2018-8-11_1.pdf	Electronic Questionnaire Survey
Repetitive Behavior Scale for Early Childhood.pdf	Electronic Questionnaire Survey
STAIAD.pdf	Electronic Questionnaire Survey
Sleep Questionnaire.docx	Electronic Questionnaire Survey
Vineland-3_Placeholder_1.png	Electronic Questionnaire Survey
Interview guide_parents_post P1P2.docx	Interview Questionnaire Survey
PEI form_PIXI.docx	Interview Questionnaire Survey
dp4_parent_caregiver_interview_response_sheet.pdf	Interview Questionnaire Survey
ASD-PEDS-Rating-Form.pdf	Observation Guide
Virtual_DAYC-2_PIXI.docx	Observation Guide
ADOS2_Placeholder.jpg	Pencil and Paper Questionnaire Survey
AOSI_Placeholder.pdf	Pencil and Paper Questionnaire Survey
BRIEF-A Self.pdf	Pencil and Paper Questionnaire Survey
Bayley-3_Placeholder.pdf	Pencil and Paper Questionnaire Survey
CSBS-DP_Placeholder.pdf	Pencil and Paper Questionnaire Survey
IBQ-R_Very Short.doc	Pencil and Paper Questionnaire Survey
M-CHAT.pdf	Pencil and Paper Questionnaire Survey
NeoEAT_Bottle_2018-11-9.pdf	Pencil and Paper Questionnaire Survey
NeoEAT_Breast_2018-11-9.pdf	Pencil and Paper Questionnaire Survey
Peabody Dev Motor_Placeholder.pdf	Pencil and Paper Questionnaire Survey
Pedi-EAT_Full_Version_2018-8-11.pdf	Pencil and Paper Questionnaire Survey
SP-2_Placeholder.png	Pencil and Paper Questionnaire Survey
Vineland-3_Placeholder.png	Pencil and Paper Questionnaire Survey
Interview_guide_parents_post_P1P2.docx	Telephone Questionnaire Survey
Generic_EI_Authorization_to_Disclose.pdf	Local Consent Form
NC EI Authorization to Disclose.docx	Local Consent Form
ECEI_Protocol Addendum Coordinating Center Lead Investigator.docx	Lead Site/Coordinating Center addendum
ASD_PEDS_VIneland_report.docx	Other
Laurie-Vismara_Standard Contract_signed.pdf	Other
PI-change-form_signed.pdf	Other
device_use_agreement_1.docx	Other
Martin___www.citiprogram.pdf	Research Ethics Training

view attachments

Addenda

Data Security Requirements

view addenda

If Principal Investigator of this study is a Student or Trainee Investigator, the Faculty Advisor certifies the following:

I accept ultimate responsibility for ensuring that this study complies with all the obligations listed above for the PI.

By certifying below, the Principal Investigator affirms the following:

I will personally conduct or supervise this research study. I will ensure that this study is performed in compliance with all applicable laws, regulations and University policies regarding human subjects research. I will obtain IRB approval before making any changes or additions to the project. I will notify the IRB of any other changes in the information provided in this application. I will provide progress reports to the IRB at least annually, or as requested. I will report promptly to the IRB all unanticipated problems or serious adverse events involving risk to human subjects. I will follow the IRB approved consent process for all subjects. I will ensure that all collaborators, students and employees assisting in this research study are informed about these obligations. All information given in this form is accurate and complete.

This study proposes research that has been determined to include Security Level 3 data security requirements. I agree to accept responsibility for managing these risks appropriately in consultation with departmental and/or campus security personnel. The Data Security Requirements addendum can be reviewed here.

Certifying Signatures:

Signature: Electronic Signature Received
Heather Hazlett

Date: 11/18/2021 04:35:30 PM
