# Supplementary material for: Pilot protocol for the Parent and Infant Inter(X)action Intervention (PIXI) feasibility study
Source: PLoS One. 2023 May 4;18(5):e0270169. doi: 10.1371/journal.pone.0270169 (PMC10159119; doi:10.1371/journal.pone.0270169)
Supplement: S1 Appendix — (DOCX) [file pone.0270169.s004.docx]

Appendix A. Social validity

1. **General**

|  | Not at all satisfied |  | Neutral |  | Very satisfied |
| --- | --- | --- | --- | --- | --- |
| 1. How satisfied are you with this intervention as a parent? | 1 | 2 | 3 | 4 | 5 |
| 1. How satisfied are you with this intervention for your baby? | 1 | 2 | 3 | 4 | 5 |

1. **Goals**

|  | Not at all applicable |  | Neutral |  | Very applicable |
| --- | --- | --- | --- | --- | --- |
| 1. How applicable were the goals of this intervention (e.g., learning about child development, fragile X syndrome, and new ways to play with baby) to you and your baby? | 1 | 2 | 3 | 4 | 5 |

1. **Procedures**

| 1. How much did you like the procedures in this intervention? | Not at all |  | Neutral |  | Very much |
| --- | --- | --- | --- | --- | --- |
|  | 1 | 2 | 3 | 4 | 5 |
| 1. How helpful was/were: | Not at all helpful |  | Neutral |  | Very Helpful |
| 1. The parent-child activities? | 1 | 2 | 3 | 4 | 5 |
| 1. The parent handouts? | 1 | 2 | 3 | 4 | 5 |
| 1. The in-home sessions? | 1 | 2 | 3 | 4 | 5 |
| 1. The telehealth sessions? | 1 | 2 | 3 | 4 | 5 |
| 1. How well did: | Not at all |  | Neutral |  | Very Well |
| 1. The interventionist listen to your concerns | 1 | 2 | 3 | 4 | 5 |
| 1. The interventionist answer your questions and/or offer suggestions? | 1 | 2 | 3 | 4 | 5 |
| 1. The intervention fit into your daily life and routines at home? | 1 | 2 | 3 | 4 | 5 |
| 1. How willing were you to carry out this intervention in general? | Not at all |  | Neutral |  | Very willing |
|  | 1 | 2 | 3 | 4 | 5 |
| 1. How reasonable was: | Not at all reasonable |  | Neutral |  | Very reasonable |
| 1. The total time commitment for you (e.g., 3–6 months total)? | 1 | 2 | 3 | 4 | 5 |
| 1. The session time commitment for you (e.g., 30–60 minutes each week)? | 1 | 2 | 3 | 4 | 5 |
| 1. How easy was it for you to be an active participant in this intervention? | Not at all easy |  | Neutral |  | Very easy |
|  | 1 | 2 | 3 | 4 | 5 |

1. **Outcomes:**

| 1. How likely is the intervention to make improvements in your child’s development? | Not at all likely |  | Neutral |  | Very likely |
| --- | --- | --- | --- | --- | --- |
|  | 1 | 2 | 3 | 4 | 5 |
| 1. How confident are you that the intervention was helpful for your child? | Not at all confident |  | Neutral |  | Very confident |
|  | 1 | 2 | 3 | 4 | 5 |
| 1. How confident are you that the intervention was helpful for you? | Not at all confident |  | Neutral |  | Very confident |
|  | 1 | 2 | 3 | 4 | 5 |

| 1. As a result of this research intervention, **I** feel: | Not True |  | Neutral |  | Very True |
| --- | --- | --- | --- | --- | --- |
| 1. Better equipped to interact with my child | 1 | 2 | 3 | 4 | 5 |
| 1. That I better understand how my child learns | 1 | 2 | 3 | 4 | 5 |
| 1. Better connected to local resources | 1 | 2 | 3 | 4 | 5 |
| 1. That the quality of my family’s life has improved | 1 | 2 | 3 | 4 | 5 |

1. **Comments:**
2. What were the most helpful parts of this intervention?
3. What were the least helpful parts of intervention?
4. What changes you’d like to see (format, length, content, interventionist style)?
5. Other comments?

Interview Guide: Parents who have completed Phases 1 and 2 of PIXI

## Introduction

Thank you for being willing to talk with us about your experience with the PIXI Intervention. Hearing about your experience will help us improve PIXI for other families whose babies are diagnosed with fragile X syndrome. So we want to hear about the things you liked, but also about any part of the intervention you didn’t like.

## Helpfulness of Intervention Components

As you know, there were several parts of PIXI. I’m going to ask you about them one-at-a-time.

During the first few sessions (when your baby was less than 6 months old), the interventionist led you in parent-child activities. The interventionist also conducted short activities with you and provided some handouts and resources on parenting and family topics.

- How did you feel about the parent-child activities that were facilitated by the interventionist? [Probes: What did you like about them? What did you not like about them? Which activities were the most/least helpful?]
- How did you feel about the handouts and resources on parenting and family topics? [Probes: How helpful were they? What topics were the most helpful? Are there other topics you think should be included in those sessions? Do you have any recommendations for improving the handouts?]

When your baby was around 6 months old, the sessions changed. The interventionist conducted weekly sessions with you about understanding your baby’s behaviors and communicating with your baby. You and the interventionist also watched videos of you interacting with your baby.

- How did you feel about the sessions on understanding your baby’s behaviors and communicating with your baby? [Probes: How helpful were they? Are there other topics you think should be included in those sessions?]
- How did you feel about reviewing the video recordings with the interventionist? [Probes: What did you like about it? What did you not like about it? How helpful was it? Would you prefer a more direct approach where the interventionist was modeling behaviors/skills and providing coaching on developing new skills or improving existing practices?]

## In person vs. Telehealth

As you know, some of the PIXI sessions were in person at your house, and some were conducted using videoconferencing on an iPad.

How did you feel about doing some sessions on the computer instead of in person? [Probes: What did you like/dislike about in-person? What did you like/dislike about videoconferencing sessions? Were there any technology challenges with the videoconferencing? DO you have any recommendations for how to improve the in-person or videoconferencing sessions?]

## Interaction with Interventionist

How was your experience with the interventionist who conducted sessions with you and assessments on your baby? [Probes: How well did she listen to your concerns? How well did she answer your questions?]

## Overall reactions to PIXI

Now I’m going to ask you some questions about your overall reactions to PIXI. Please think about all the different parts of PIXI when responding.

- How, if at all, do you think PIXI helped your baby? IF so, in what ways?
- How, if at all, do you think PIXI helped you? If so, in what ways?
- What were the most helpful parts of PIXI?
- What were the least helpful parts of PIXI?
- How easy or hard was it for you to participate in this intervention? [Probes: What would make it easier to participate?]
- What, if anything, would you change about the PIXI Intervention? [Probes: Format, length, content, interventionist style]
- Is there anything specific you would have liked to have seen included as part of the PIXI intervention?

## Closing

Thank you again for your time!
